# Random forests for survival data: which methods work best and under what conditions?

**DOI:** 10.1515/ijb-2023-0056

**Published:** 2024-04-24

**Authors:** Matthew Berkowitz, Rachel MacKay Altman, Thomas M. Loughin

**Affiliations:** Statistics and Actuarial Science, Simon Fraser University, Burnaby, Canada

**Keywords:** random survival forest, random forest, survival analysis, point prediction, survival function estimation

## Abstract

Few systematic comparisons of methods for constructing survival trees and forests exist in the literature. Importantly, when the goal is to predict a survival time or estimate a survival function, the optimal choice of method is unclear. We use an extensive simulation study to systematically investigate various factors that influence survival forest performance – forest construction method, censoring, sample size, distribution of the response, structure of the linear predictor, and presence of correlated or noisy covariates. In particular, we study 11 methods that have recently been proposed in the literature and identify 6 top performers. We find that all the factors that we investigate have significant impact on the methods’ relative accuracy of point predictions of survival times and survival function estimates. We use our results to make recommendations for which methods to use in a given context and offer explanations for the observed differences in relative performance.

## 1 Introduction

Survival analysis – the statistical analysis of event times like deaths or disease recurrence – is important for many studies in medicine, as well as in manufacturing and engineering [1]. Parametric analysis of survival data is well established [1] and can easily achieve the dual goals of estimating a probability of survival beyond some fixed time *t* and predicting the survival time for a subject with a particular set of covariates. However, as with any other parametric statistical analysis, the validity of the results depends, sometimes critically, on how well the proposed model approximates the true distribution of responses. Random forests for regression and classification [2] are able to detect both direct and interacting signals from covariates, and they can estimate complex regression surfaces from arbitrary response distributions without requiring that a model be specified. These remarkable features have attracted researchers to adapt random forests to handle the complexities imposed by survival data, which are usually censored and highly skewed. Random forests for survival data and related techniques have been developed that produce estimated survival distributions and/or predicted survival times using ensembles of trees that are built and processed in varying ways [3], [4], [5], [6], [7], [8], [9], [10], [11], [12], [13], [14], [15].

Given the growing literature on survival trees and forests, knowing which techniques perform well in terms of point prediction and/or survival function estimation is important. However, few systematic comparisons of the performance of survival trees and forests have been conducted.

An earlier study by Radespiel-Tröger et al. [16] compared seven splitting criteria for building individual survival trees (not forests). They concluded, based on an application to a single real dataset, that splits using an adjusted log-rank statistic produce the best results using the integrated Brier score (IBS) and a version of *R* ^2^. Later, Shimokawa et al. [17] compared nine older (proposed before 2000) splitting criteria for individual survival trees through a simulation study. They compared four data-generating mechanisms, each at four censoring rates, and evaluated the performance of each splitting criterion using IBS and a form of *R* ^2^. They found that all but two methods performed comparably well in general at all levels of censoring, and recommended certain splitting criteria over others depending on the data setting (i.e., censoring rate and shape of hazard rate).

Moradian et al. [10] compared seven methods for building survival forests against two standard, non-forest methods using simulation studies and real datasets. The authors tested these against two benchmarks: a Cox model and the Kaplan–Meier (K-M) estimator. They found that their proposed splitting criteria tended to rank among the best performers according to the integrated absolute error and IBS metrics, with much more variable rankings according to the C-index metric. Our simulation study, in contrast, is much more extensive in that it tests various factors that affect survival forest performance.

To our knowledge, to date, no one has undertaken an in-depth comparison of the many alternative forest-based methods for survival analysis. In light of the fact that they are based on a variety of algorithms that focus on producing estimates of different population quantities, these methods may perform quite differently for different tasks. The lack of systematic comparison leaves a knowledge void for both practitioners and researchers. With this paper, we aim to fill part of this gap and to supply both practitioners and statistics and machine learning researchers with advice on which techniques should be considered for the analysis of survival data with particular properties. We systematically investigated various factors that influence survival forest performance (forest construction method, censoring, sample size, distribution of the response, structure of the linear predictor, and presence of correlated or noisy covariates via an extensive simulation study with well-defined performance metrics. In particular, we studied 11 methods that have recently been proposed in the literature and identified 6 top performers. We found that all the factors that we investigated have significant impact on the methods’ relative accuracy of point predictions of survival times and survival function estimates.

We begin in Section 2 with a review of more recently proposed methods for building survival forests. In Section 3, we describe in detail the design of our simulation study as well as the metrics we used to evaluate performance. In Section 4, we discuss our results and recommendations for practitioners. In Section 5, we offer explanations for the observed differences in some methods’ relative performance in certain settings. We conclude with our main contributions, our study’s limitations, and future research opportunities in Section 6.

## 2 Survival forest methods review

Our review of the literature revealed many methods for building forest-based ensembles for survival data (hereafter “survival forests”). For our comparative study, we focused on more recent methods because they are generally favoured in other papers’ comparisons. We also restricted our focus to methods designed for estimating survival functions and point prediction. Therefore, we excluded methods focused on estimating treatment effects (e.g., [18, 19]). Table 1 lists all such methods proposed since 2000 that we were able to find. The first five methods are based on the forest construction algorithm proposed by Ishwaran et al. [6] and differ only in the splitting criterion used (thus we name the methods based on the splitting criterion). The other methods, proposed in mostly subsequent papers, are characterized by alternative forest construction algorithms that differ in ways beyond the splitting criterion. We describe all these methods below.

**Table 1:** Survival forest methods compared in simulation study.

Method	Source
Log-rank (RSF-log-rank)	[6]
Log-rank score (RSF-log-rank-score)	[6]
C-index (RSF-C-index)	[9]
*L*_1_	[10]
Brier score (RSF-Brier-score)	[11, 12]
Conditional inference forest (CIF)	[4]
Recursively imputed survival trees (RIST)	[7]
Rotational survival forest (RotSF)	[8]
Oblique random survival forest (ORSF)	[13]
Censored generalized random forest (CRF-GRF)	[14, 15]
Censored quantile regression forest (CRF-QRF)	[5, 15]

The most popular method, *random survival forests* (RSFs), proposed by Ishwaran et al. [6], adapts the random forest algorithm [2] to survival data. The main algorithmic adaptations in random survival forests are the splitting criterion used to recursively partition the bootstrap samples and the statistic that summarizes the observations in each terminal node of each tree in the ensemble.

The RSF algorithm begins by drawing *B* bootstrap samples from the training data. The data excluded from each sample represent a test set, the “out-of-bag” (OOB) data, for each sample. A survival tree is then constructed using each bootstrap sample. At each tree node, *M* covariates are randomly selected as candidates upon which to base a split. The split is made on the candidate variable that results in the greatest survival differences, according to some splitting criterion, between the observations in each child node. Successive splits are then made on each of these child nodes until the tree is grown to full size, ensuring that each terminal node contains no fewer than one unique failure. The observations in each terminal node are then used to compute the Nelson-Aalen estimate of the cumulative hazard function (CHF) for that node. Therefore, for a new individual, each tree produces an estimated CHF. These estimates can then be averaged to obtain an ensemble CHF, which serves as the final RSF prediction for this individual [6].

Ishwaran et al. [6] proposed several splitting criteria for constructing trees within RSFs. The *log-rank* splitting criterion, which finds the split that maximizes the log-rank statistic, has since become perhaps the standard splitting criterion. Ishwaran et al. [6] proposed a standardized variant of the log-rank splitting criterion, the *log-rank score* statistic.

Other statistics have been proposed by other authors as splitting criteria for RSFs. Schmid et al. [9] proposed the C-index, hypothesizing that better performance might be achieved when the same method is used for both splitting and for measuring error (C-index is the standard method of quantifying prediction error of RSFs, which we describe in more detail in Section 3.3.1). Due to the suboptimal performance of the log-rank splitting criterion when survival or hazard functions cross, Moradian et al. [10] proposed an *L*_1_ splitting criterion that is based on the integrated absolute difference between each child node’s estimated survival distributions. Boström et al. [11, 12] used the Brier score – another commonly used error metric (see Section 3.3.2) – as a splitting criterion. We refer to the forests based on the three splitting criteria discussed in this paragraph as RSF-C-index, *L*_1_, and RSF-Brier.

Random forests have a bias towards variables with many possible splits, as identified by Breiman et al. [20]. To address this issue, Hothorn et al. [4] proposed a new type of decision tree, termed a “conditional inference” tree, which can be amalgamated into conditional inference forests (CIFs). CIF-based trees differ from RSF-based trees in several key ways. First, after randomly selecting *M* covariates (similarly to the RSF algorithm), a null hypothesis of independence between each candidate variable and the survival outcome is tested using permutation tests [21]. The candidate variable with the strongest association to the survival outcome is chosen for splitting. If there is no statistical evidence of an association between the survival outcome and any of the candidate variables, the node becomes a terminal node. Second, the CIF differs from the RSF not just in the structure of individual trees but also in the aggregation mechanism. Specifically, while RSFs use an equal weighting scheme to compute the ensemble CHF, CIFs adopt a nearest neighbour weighting scheme to aggregate observation times from each tree, yielding an ensemble survival function.

The *k*-fold *RIST* algorithm [7] is based on the extremely randomized trees (ERTs) algorithm [22]. To start, the algorithm generates *B* ERTs. For each node split, a random subset of covariates is selected (like with RSFs); for each of these covariates, a random split point is proposed, and the one that best optimizes a specific criterion is chosen for the actual split. The ERTs are then aggregated to estimate the survival distribution for each censored observation (given the covariates). Using these distributions to impute *B* sets of random survival times with which to replace the censored survival times, *B* new datasets are created and *B* new ERTs are fit. This process of model fitting and imputation is recursively repeated *k* ≥ 1 times, where *k* is selected by the user. The survival distribution for a new individual is estimated via the ensemble survival function based on the ERTs in the *k*th iteration.

The *RotSF* algorithm produces an ensemble of trees, where each tree is based on a bootstrapped sample and a perturbed version of the matrix of explanatory variables [8]. The perturbations are achieved by applying principal components rotations to randomly selected subsets of explanatory variables separately for each tree. The standard RSF algorithm is then applied to the resulting dataset.

*ORSF* also transforms the matrix of explanatory variables used for building trees [13]. At each split, Cox proportional hazards models are fit using three different amounts of regularization. These fitted models produce three estimates of the hazard function for each individual. These estimated hazard functions – which are proportional to exponentiated linear combinations of the variables selected for that split – act as three candidate splitting variables. The log-rank splitting criterion is then used in the usual manner to determine whether the node should be split on any of these variables and, if so, where. ORSF requires selecting the tuning parameter, *α*, which governs the elastic net penalty [13]; Zou and Hastie [23] found that the choice of *α* = 0.05, indicating an elastic ridge-type penalty, led to the “best” performance according to their error metrics. Like CIF, ORSF produces a weighted K-M estimate of the conditional survival function instead of a CHF, and it uses sampling without replacement instead of bootstrap sampling.

Li and Bradic [15] proposed *censored regression forests* based on a simple relationship among the conditional distributions of the observed times (the minimum of the survival and censoring times), the survival times, and the censoring times. Their algorithm involves first estimating the conditional distribution of the observed times, given the covariates, using either generalized random forests (CRF-GRF; [14]) or quantile regression forests (CRF-QRF; [5]). They then estimate the conditional distribution of the *censoring* times given the covariates (via, for example, the K-M estimate). Conditional quantiles of the distribution of survival times are then estimated from the quotient of these two estimated conditional distributions.

## 3 Methods

In this section, we describe the design of our simulation study, which we used to compare survival forest methods, and the error metrics we used to evaluate each method’s performance.

We use the following notation throughout. Let *T* and *C* be the survival time and censoring time, respectively, of an individual. We assume that *T* and *C* are conditionally independent given the covariates. Let *Y* = min(*T*, *C*) be the observed time, and let δ=1(T≤C) denote the censoring indicator. Let **X** = (*X*_1_, …, *X*_*p*_) denote a *p*-dimensional vector of covariates. Let *S*(*t*) and *h*(*t*) denote the survival and hazard functions, respectively, of *T*. We assume in all cases that the covariates affect the conditional distribution of survival times only through a linear predictor, ***Xβ***, where ***β*** is a vector of parameters that modifies the effect of ***X***. We describe the linear predictors that we consider in Section 3.1.2.

### 3.1 Factors in the Simulation Study

Our review of the literature revealed several important factors that contribute to the relative performance of some survival tree and forest methods. As we discuss in the following subsections, these factors include the distribution of the response, the structure of the linear predictor, the censoring rate, the size of the training sample, and the presence of noisy or correlated variables. We also discuss our chosen levels of these factors. Our simulation study investigates how survival forest methods perform across the combinations of these factor levels – hereafter called factor-level combinations (FLCs).

#### 3.1.1 Methods for building survival forests

The first factor in our simulation study is the analysis method used for building survival forests, henceforth denoted as Method. In Section 2, we reviewed every method proposed (to our knowledge) in the more recent survival forest literature. For our simulation study, we compared twelve methods (the eleven methods in Table 1, with two versions of RIST) for building survival forests under various simulation settings. We used each method “out of the box”, i.e., as their inventors proposed them in their original papers, using default values for all tuning parameters.

All methods except for *L*_1_, CRF-GRF, and CRF-QRF provide estimates of the individuals’ survival functions in the same format; specifically, the output is a matrix with entry (*i*, *j*) corresponding to the estimated value of the *i*th individual’s survival function at the *j*th ordered, observed survival time in the training sample. The output of the three exception methods consists of much shorter vectors of survival function estimates for each individual, in particular, estimates at only a subset of the observed survival times. This difference will become relevant when considering error metrics for evaluating forest performance in Section 3.3.

#### 3.1.2 Data-generating mechanisms

The second and third factors in our study are the data-generating mechanisms (DGMs), both with respect to the distribution of the response (DGM-Y) and the structure of the linear predictor (DGM-X). Based on results from the literature, we expect certain methods to work better under different simulation settings. For example, some methods appear to have a comparative advantage when the survival or hazard functions for different levels of categorical covariates cross (e.g., [10]). Others appear to perform well across a variety of data settings (e.g., [7]). Yet the impact of DGMs on method performance has not been studied in a comprehensive, systematic way. Therefore, we included a wide variety of DGM-Ys and DGM-Xs as factors in our study.

##### 3.1.2.1 DGM-Y

We included three DGM-Ys representing different properties that are common in models for survival distributions. Our first DGM-Y is the Weibull distribution parameterized to be a type of proportional hazards model. We refer to this DGM-Y as “Y-Weibull”. The hazard function associated with this model is

$$\begin{array}{ll} h\left(t|x\right) & =\lambda \rho t^{\rho -1}\exp\left(x^{\prime }\beta \right)\\ & \equiv h_{0}\left(t\right)\exp\left(x^{\prime }\beta \right), \end{array}$$

where *ρ* and *λ* are the shape and scale parameters, respectively, of the Weibull distribution, *h*_0_(*t*) is the baseline hazard function, and ***β*** excludes the intercept. This parameterization is consistent with that of Cox proportional hazards models.

Our second DGM-Y is the lognormal distribution parameterized to be a type of accelerated failure time (AFT) model. We refer to this DGM-Y as “Y-AFT”. More specifically, we assumed the relationship

$$log\left(t\right)=x^{\prime }\beta +\epsilon ,$$

where *ϵ* ∼ *N*(0, *σ* ^2^). In this model, ***β*** *does* contain an intercept term. The associated hazard function is

$$\begin{array}{ll} h\left(t|x\right) & =\lambda \rho {\left(t\cdot \exp\left\{x^{\prime }\beta \right\}\right)}^{\rho -1}\exp\left(x^{\prime }\beta \right)\\ & \equiv h_{0}\left(t\cdot \exp\left\{x^{\prime }\beta \right\}\right)\exp\left(x^{\prime }\beta \right). \end{array}$$

Note that Y-AFT can have crossing survival curves but not crossing hazards [24].

Our third DGM-Y, which we call “Y-GG”, is the generalized gamma distribution parameterized to allow not only non-proportional hazard functions but also intersecting survival and hazard functions. The inclusion of this DGM-Y is due to concerns in the literature that some survival forest methods do not perform well when hazards cross [10, 17]. The generalized gamma density is

$$f\left(t|x\right)=\frac{\left(\frac{b}{a^{\prime bk}}\right)t^{bk-1}\exp{\left(-t/a^{\prime }\right)}^{b}}{\Gamma \left(k\right)},$$

where *a*′ = *a* ⋅ exp(**x′*β***) is a scale parameter, and *b* and *k* are both shape parameters. The generalized gamma distribution *GG*(*a*, *b*, 1) (i.e., the case where *k* = 1) is equivalent to the Weibull (*a*, *b*) distribution. As in the Y-Weibull case, ***β*** does not contain an intercept term. The associated hazard function is

$$h\left(t|x\right)=\frac{\left(\frac{b}{a^{\prime bk}}\right)t^{bk-1}\exp{\left(-t/a^{\prime }\right)}^{b}}{\Gamma \left(k\right)-\gamma \left(k,{\left\{t/d\right\}}^{b}\right)},$$

where *γ*(⋅) denotes the lower incomplete gamma function.

To produce crossing hazards using the generalized gamma distribution, we used two different values of *k*. In particular, for half the samples within each dataset, indicated by the levels of the covariate *X*_1_ described in the next subsection, we set *k* = 1/16 to produce a bathtub-shaped hazard, and for the other half, we set *k* = 1 to produce a Weibull (monotonic) hazard function that intersects with the hazard (and survival) function of the first half of the sample. This approach was inspired by [25], who used two different families of distributions to generate non-proportional hazards.

To generate draws from each DGM-Y, we used the inverse cdf evaluated at random draws from a *U*(0, 1) distribution.

##### 3.1.2.2 DGM-X

Each DGM-X is a function of 10 independent covariates, *X*_1_, …, *X*_10_. The covariate *X*_1_ has a Ber(0.5) distribution. Five covariates are uniform variables, i.e., *X*_2_, …, *X*_6_ ∼ *U*(0, 1). The remaining covariates are five-level categorical variables, i.e., *X*_7_, …, *X*_10_ ∼Multinomial(*p*_*i*_), with *p*_*i*_ ≥ 0 and ∑*p*_*i*_ = 1.

We included four different DGM-Xs. In the first DGM-X, the covariates appear as linear terms in the linear predictor, ***Xβ***. We refer to this DGM-X as “X-baseline”. Note that in Y-Weibull, X-baseline has a linear relationship with the log hazard; in Y-AFT and Y-GG, X-baseline has a linear relationship with log mean survival time.

In our second DGM-X, four numeric terms from X-baseline are replaced with polynomial and/or interaction terms, and one additional interaction term is added. The interaction terms involve only numeric covariates. We refer to this DGM-X as “X-poly-int”.

In the third DGM-X, a sine transformation is applied to one numeric covariate in X-baseline. This function mimics seasonality that may occur in some time-to-event scenarios. We refer to this DGM-X as “X-sine”.

In the fourth DGM-X (“X-piecewise”), two of the covariates from X-baseline are allowed to have different coefficients depending on their values. The range of each covariate is split into three pieces, and a different parameter is assigned to each segment. The functions within each segment are linear.

The rationale for the inclusion of these latter three DGM-Xs was that preliminary findings in the literature show that some survival forest methods may not be as effective as others at handling such relationships (e.g., [10, 17]).

Refer to Appendix C for more specifics about the structure of the linear predictors in each DGM-X.

#### 3.1.3 Censoring rate

An important determinant of the relative success of some forest construction methods is the proportion of censored observations. For example, both the RSF-C-index and the *L*_1_ splitting criteria are suspected to perform relatively well with higher censoring rates [9, 10]. Therefore, our experimental design considered three levels of censoring, 10 %, 25 %, and 70 %, representing relatively light, moderate, and relatively heavy censoring, respectively. We exclude the no-censoring scenario because these methods were not designed for cases where the survival times are fully observed. For each DGM, censoring times were independent with identical exponential distributions. The rate parameters were chosen to give the desired censoring proportion (on average across datasets within DGM).

#### 3.1.4 Training sample size

The sample size of the training set is another likely important variable that affects the performance of the methods. We selected two levels, *n*_1_ = 200 and *n*_2_ = 1500, to compare whether some methods perform relatively better than other methods with smaller or larger sample sizes. These two sample sizes reflect the range of survival dataset sizes we have encountered in practice.

#### 3.1.5 Extra variables

Our final factor, “extra variables”, concerns the addition of unimportant covariates in the data. We considered three levels of this factor: the “baseline level” (no extra covariates); the “correlated level” (three additional mean zero normal variables that are non-causally associated with the response and are highly correlated [*ρ* = 0.75] with an important covariate); and the “noise level” (50 additional unimportant, standard normal random variables – 30 that had pairwise correlations of 0.5 with one another but were independent of all other covariates and 20 that were independent of each other and all other covariates).

### 3.2 Signal-to-noise ratio

Changing levels of some factors can create models with greater or lesser association between the survival times and the linear predictors. The effect of this association is thus confounded with the effects of the factors we are changing experimentally. To eliminate this confounding, we enforced an approximately constant signal-to-noise ratio (SNR) across FLCs. To quantify the SNR, we adapted the standard SNR for linear regression [26]. We first defined a pseudo *R* ^2^,

$$R^{2}=\frac{\mathrm{Var}\left(E\left(T|X\right)\right)}{\mathrm{Var}\left(E\left(T|X\right)\right)+E\left(\mathrm{Var}\left(T|X\right)\right)},$$

where the outer expectations and variances are taken with respect to the distribution of *X*. Analogous to the standard *R* ^2^ in regression, the pseudo *R* ^2^ represents the fraction of total variability in the marginal distribution of survival times that is attributable to changes in *X* as opposed to inherent variability related to generating survival times conditional on *X*. We then defined the SNR as

$$\text{SNR}=\frac{R^{2}}{1-R^{2}}.$$

We controlled the SNR across FLCs by simulating a large number of observations at each FLC, then adjusting the scale and regression parameters to achieve approximate equality across each of these preliminary simulations.

Refer to Appendix A for more details, including the conditional mean and variance of the response variables in each of our three DGM-Ys.

### 3.3 Responses of interest: error metrics

As explained in Section 3.1.1, the output of each method is an estimated survival function for each individual, from which we can extract an estimated median. One quantity of interest is the distance between individuals’ estimated and true survival functions. Another interest is the closeness of the estimated medians (which we use as point predictions) to the observed times. We treat these two types of errors as the responses in our simulation study.

Our rationale for distinguishing between point prediction and survival function estimation is that, in some clinical settings, practitioners are mainly interested in predicting a specific survival time (e.g., for a cancer patient [27, 28]). In other settings, researchers may be concerned with the survival function (e.g., for the purpose of characterizing the probability of a machine part’s malfunctioning over a given time frame [29]).

We use error metrics that are specifically designed for comparing distributions or point predictions. For the simulation study, for point prediction, we use absolute loss and Harrell’s concordance index (C-index); for survival function estimation, we use the integrated Brier score (IBS). We briefly discuss each below.

Let *t*_*i*_ and *c*_*i*_ denote realizations of the survival and censoring times, respectively, for subject *i*, *i* = 1, …, *n*. Let *y*_*i*_ = min(*t*_*i*_, *c*_*i*_) be the observed time, and let $\delta_{i}=1\left(t_{i}\leq c_{i}\right)$ denote the observed censoring indicator. Let **x**_*i*_ = (*x*_*i*1_, …, *x*_*ip*_) denote the observed covariates for the *i*th individual. Thus, the observed data are denoted by (*y*_*i*_, *δ*_*i*_, **x**_*i*_).

We used test set sample sizes of n* = 200 to evaluate the performance of each method applied to each dataset generated from each FLC.

#### 3.3.1 Absolute loss and C-index

Because the survival times, *t*_*i*_, *i* = 1, …, *n*, are available in the simulated data, we can use an evaluation metric that compares the predicted survival times with these survival times. The absolute loss is defined as

$$L=\frac{1}{n}\overset{n}{\underset{i=1}{\sum }}|t_{i}-{\hat{m}}_{i}|,$$

where ${\hat{m}}_{i}$ is the estimated median survival time at **x**_*i*_. When the estimated survival curve does not reach 0.5, the estimated median – and thus *L* – are unavailable, a weakness of the measure. In this case, we use the estimate of the maximum quantile that is available [30]. However, under our settings, we can normally obtain an estimate of the median.

In practice, when some survival times are unobserved, computing absolute loss is not possible. We therefore need an alternative error metric that accounts for censoring. Ishwaran et al. [6] proposed using Harrell’s concordance index (C-index) as a metric of error to evaluate the fit of RSFs [31]. The C-index is a function of the ranks of the observed survival and censoring times and the ranks of the predicted outcomes. Whereas Ishwaran et al. [6] chose the “predicted mortality” – defined as the ensemble CHF for the *i*th individual summed over all survival times – as the predicted outcome, we choose the estimated median survival time, ${\hat{m}}_{i}$ (or maximum available estimated quantile, as per the previous paragraph). So for our case, individual *i* has a longer predicted survival time than individual *j* if ${\hat{m}}_{i}>{\hat{m}}_{j}$ .

The C-index is an estimate of the probability that the order of two predicted survival times in a randomly selected pair of individuals has the same order as the actual survival times [6]. To compute the C-index, we use the following steps:

(i) Form all possible pairs of observed times (*y*_*i*_, *y*_*j*_), 1 ≤ *i* < *j* ≤ *n* from the entire dataset.
(ii) Omit pairs where the shorter observed time is censored, or where the observed times are equal and both censored. Denote the remaining pairs as *permissible.*
(iii) If *y*_*i*_ ≠ *y*_*j*_, count 1 if the shorter observed time had a shorter predicted outcome; count 0.5 if ${\hat{m}}_{i}={\hat{m}}_{j}$ .
(iv) If *y*_*i*_ = *y*_*j*_, count 1 if ${\hat{m}}_{i}={\hat{m}}_{j}$ ; count 0.5 if ${\hat{m}}_{i}\neq {\hat{m}}_{j}$ .
(v) The *concordance* is the sum of the counts over all permissible pairs.

The C-index, *C*, is then defined as the ratio of the concordance to the number of permissible pairs. We define the prediction error based on the C-index as *E* = 1 − *C*, where 0 ≤ *E* ≤ 1. Under this definition, *E* = 0 indicates perfect prediction of the ordering of survival times, and *E* = 0.5 indicates performance equivalent to random prediction. We use *E* so that a lower error implies better performance in the case of both point prediction error metrics.

Absolute loss and the C-index each have advantages and disadvantages. Unlike absolute loss, the C-index can be computed even when some survival times are censored. However, the C-index measures only whether the predicted responses are ordered similarly to the observed responses, not whether they are accurate estimates. In contrast, our main interest is in comparing the accuracy of predictions produced by different methods. Ideally, absolute loss and the C-index would lead to the same conclusions about overall method performance, but sometimes they do not (see Section 4). Ultimately, we prioritize absolute loss for assessing point predictions because it directly measures estimation accuracy, but we also briefly consider the C-index when discussing our results and making recommendations.

#### 3.3.2 Integrated Brier score

The Brier score is one of the most widely used metrics to quantify the error in an estimated survival distribution, $\hat{S}\left(t|x_{i}\right)$ [32, 33]. The Brier score (BS) for the survival status of the *i*th individual at time *t* is defined as

$${\text{BS}}_{i}\left(t\right)={\left[1\left(y_{i}>t\right)-\hat{S}\left(t|x_{i}\right)\right]}^{2}.$$

The integrated Brier score (IBS) is defined as

$$\text{IBS}=\frac{1}{n}\overset{n}{\underset{i=1}{\sum }}\frac{1}{w}\overset{w}{\underset{0}{\int }}BS_{i}\left(t\right)dt,$$

where *w* = max_*j*_ *y*_*j*_. While most forest methods provide an estimate of the conditional survival function at each observed survival time, *L*_1_, CRF-GRF, and CRF-QRF provide an estimate only at a subset of these times. Therefore, the survival function estimates based on these methods are comparatively coarse, and the associated IBS values are comparatively huge. For this reason, we exclude these methods from comparisons using IBS.

### 3.4 Pilot versus comprehensive simulation study

Testing every combination of factor levels described in Section 3.1 would involve 3 × 4 × 3 × 2 × 3 = 216 FLCs, with each method applied to each FLC. Because performing all 12 analysis methods on 216 FLCs would be an extensive and time-consuming undertaking, we first performed a pilot study to determine the relative ability of our chosen factor levels to differentiate performance of the methods. For the pilot study, we selected a subset of these FLCs so that we could obtain unbiased estimates of all main effects and two-way interactions among data-generating factors and their three-way interactions with Method (our factor of primary interest). We then applied all 12 methods to these combinations. The result was an experiment using only 60 FLCs that allowed unbiased estimation of all main effects and many two-way interaction effects and corresponding interactions with Method. To account for inherent variability in performance, we generated *R* = 10 datasets from each FLC and applied each method in Table 1 to each dataset.

For our comprehensive study, we assessed only the most competitive methods and retained only the most important factors and factor levels, as identified by the pilot study. We then included every FLC among the remaining factors and factor levels (again using 10 replicates per FLC) and applied each selected method to all datasets. Our final design involves 3 × 4 × 2 × 2 × 3 = 144 FLCs and 6 methods. Note that using 10 replicates within each FLC results in 1440 observations in total. Comparisons of means at two levels of any factor use a substantial fraction of these observations in each comparison, increasing the power to detect meaningful differences.

Table 2 summarizes the different factors and their levels.

**Table 2:** Factor levels used in our pilot and comprehensive studies; starred levels were excluded in the comprehensive study.

Factor					
Method	DGM-Y	DGM-X	Censoring	Extra variables	Training sample size
CIF	Weibull	Baseline	10 %	0	200
RSF-log-rank	AFT	Poly-int	25 %*	3 (correlated)	1500
RSF-log-rank-score*	GG	Sine	70 %	50 (noise)	
RIST-1		Piecewise			
RIST-3*					
RotSF					
RSF-C-index*					
*L*_1_					
RSF-Brier-score*					
ORSF					
CRF-GRF*					
CRF-QRF*					

Please see Appendix F for a more technical discussion of our experimental design.

### 3.5 Analysis of factorial experiment

To identify potentially important factors and factor levels, we fit linear mixed models (LMMs) using each of three response variables: transformed absolute loss, transformed IBS, and C-index. The models included treatment contrasts for all main effects, all two-way interactions, and any three-way interactions in which Method was one of the factors. We included a dataset-specific random effect to account for the repeated measures since we applied every survival forest method to every generated dataset. Note that we excluded three methods (*L*_1_, CRF-GRF, and CRF-QRF) from the model in which IBS was the response variable because the output that these methods produce does not allow for the IBS to be computed in a way that is comparable to the other methods (see Section 3.3.2).

To better satisfy the normality and constant variance assumptions of the LMM, we used log(IBS) and log(log(absolute loss)) as the responses in the first two aforementioned models. We computed the ANOVA table based on the marginal (i.e., not conditional on the random effect) Type III sums of squares and then sorted the F-values to identify the effects of greatest magnitude (and, ultimately, the most important factors, conditional on including all other factors in the model). We used our findings to guide the subset of results we present in Section 4.3. We also examined interaction plots constructed from these LMMs to identify the most important factor levels, which we discuss in Section 4.4.

### 3.6 Implementation

The majority of the selected methods have R packages built for their implementation. RSF-log-rank, RSF-log-rank -score, and RSF-Brier-score are all built into the randomForestSRC package. RSF-C-index can be implemented using the ranger package [34]. CIF can be fit using the party package. ORSF can be implemented using the aorsf package. CRFs can be built using code from the GitHub page of one author [30] in conjunction with the grf package. Similarly, RotSF can be built using code from an author’s GitHub page [35]. Lastly, we obtained code from one of the authors of [10] to implement the RSF method with the *L*_1_ splitting criterion. We have also made all code available for this paper on the primary author's GitHub page: https://github.com/mcberko/survivalforests.

## 4 Results

We begin by giving an overview of our results. We present the details in Sections 4.1 and 4.2, followed by formal recommendations in Section 4.3. An even more granular discussion follows in Section 4.4, where we focus on the most influential factors and factor combinations. We present results mainly from the comprehensive study, but we also comment on the pilot study where relevant.

Based on the totality of the results from our studies, all six methods included in our comprehensive study were relatively competitive overall, with generally good performance across most FLCs and rare poor performance. As detailed below, some methods (notably, RotSF and RIST) performed especially well in certain settings, while others (e.g., RSF-log-rank) performed more consistently below average relative to the six top-performing methods.

Our pilot study revealed that RSF-log-rank-score was clearly the worst-performing method and also that CRF-GRF, CRF-QRF, RSF-Brier, and RSF-C-index were relatively uncompetitive both for point prediction and for survival function estimation (see Supplementary Information for relevant plots).

For the ensuing discussion, we separate our recommendations of methods for point prediction and those for survival function estimation based on the rationale provided in Section 3.3; that is, in some clinical settings, practitioners are likely more concerned with predicting a survival time, whereas in other settings, the entire survival function will be of interest.

### 4.1 Descriptive statistics

**Figure 1:**
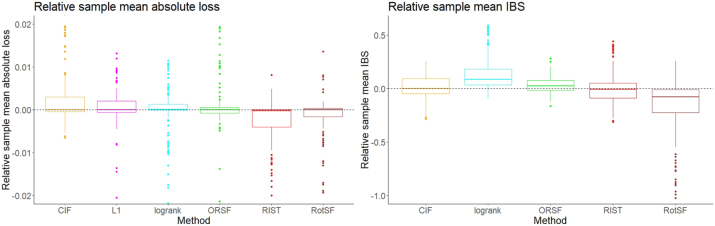
Relative sample mean absolute loss by method (left); relative sample mean IBS by method.

In Figure 1, we present boxplots of relative sample mean error values. The boxplots are comparisons of methods’ relative performance across all 144 FLCs in the comprehensive simulation study. Specifically, for each error metric, we computed the sample mean error across the 10 replicate datasets for each method separately within each FLC. We then centred these sample means around the overall sample mean (across methods) for that FLC. Negative values therefore represent better observed performance (smaller average error) for that FLC. Each boxplot consists of 144 relative mean error estimates.

We emphasize that we present the boxplots to summarize and visualize differences in method performance across FLCs. We conduct formal inference in Section 4.2.

#### 4.1.1 Point prediction

We used the estimated median for point prediction of survival time, and we quantified the accuracy of the predictions using absolute loss and the C-index (as discussed in Section 3.3.1).

In the sample from our comprehensive study, the median performances of all six methods were comparable, while the variability and distributions differed (see left panel of Figure 1). Using a few criteria, we can identify better and worse methods. Specifically, RIST had absolute loss below the mean for at least 3/4 of the FLCs (upper limit of box is below 0). All other methods – especially, CIF, *L*_1_, and RSF-log-rank – frequently had absolute losses above the mean, with their rather variable upper quartiles entirely above zero, although their medians occur essentially at the mean among methods. The tails at one or both ends of each boxplot are long, which may have several causes. It may relate to relative ease or difficulty that different methods have in producing accurate survival time predictions under different FLCs. It may also reflect the variability of absolute loss, and more generally the difficulty in doing point prediction accurately using any method in any situation compared to survival function estimation – see next section.

Somewhat different results emerged using *E* = 1 − *C* (see Figure 12 in Appendix B). Unlike with absolute loss, CIF and ORSF more often performed better than the mean according to *E*. The other methods’ relative performance was similar using both point prediction error metrics.

Within each method and each FLC, the sample correlations of the two error metrics averaged roughly −0.11 but varied from −0.53 to 0.45. Since the two metrics represent different underlying quantities, divergence in performance is not necessarily concerning or surprising. Simple scenarios involving observed and predicted survival times can be constructed such that the two metrics yield opposite conclusions regarding which method is “best”. For example, the predicted rank could be perfect, yet the absolute differences could be relatively large; or the predicted rank could be terrible, yet the absolute differences could be relatively tiny. Clearly, *E* cannot be used as a surrogate for absolute loss. Therefore, we caution against using *E* when the specific goal is minimizing the absolute difference between predicted and true survival times. It is an appropriate measure only when predicting rank or ordering of survival times is the primary interest. See the Supplementary Information for histograms of the sample correlations between the two error metrics.

#### 4.1.2 Survival function estimation

For survival function estimation, in the sample from our comprehensive study, RotSF had estimated mean IBS that was lower than the average for all methods for at least 3/4 of all FLCs (see right panel of Figure 1). Conversely, RSF-log-rank performed worse than average for over 3/4 of all FLCs. The other three methods, CIF, ORSF, and RIST, performed roughly similarly in both median performance and variability, with RIST performing relatively badly in some outlying cases. Recall that we excluded *L*_1_ (as well as CRF-GRF and CRF-QRF in the pilot study) due to difficulties in computing IBS from their output (see Section 3.3.2).

### 4.2 Effects of sample size and censoring

In this subsection, we examine methods in more detail for specific combinations of the Censoring and SampleSize factors because they influence method performance and are known (or can be trivially estimated) prior to the analysis of a dataset. The other factors, DGM-Y, DGM-X, and ExtraVars, while possibly important, are hard to determine from the data (though subject-area knowledge may provide some information). We acknowledge that performance differences among the combinations of Censoring and SampleSize levels can be influenced by differences in their effects at the levels of other factors. But we want to present results for guidance in the common case where the analyst is blind to the levels of these factors in their study population. We defer to Section 4.4 discussion of how these more elusive factors affect method performance.

As in Section 4.1, we separate our recommendations for point prediction and survival function estimation. In each subsection, we discuss relative performance differences of the methods for each combination of Censoring and SampleSize, then summarize the findings in Table 3 (in Section 4.3).

**Table 3:** The better-than-average (“+”) and worse-than-average (“−”) performing survival forest construction methods for each combination of Censoring (*C*) and SampleSize (*n*), for each error metric.

Loss	*C*	*n*	CIF	ORSF	RIST-1	RotSF	RSF-log-rank	L1
AL	10	200	−	−	+	+	+	−
	10	1500	−	−	+	−	+	+
	70	200						
	70	1500					−	
IBS	10	200	−		+	+		
	10	1500	−	+		−	−	
	70	200		−	−	+	−	
	70	1500	+	−	−	+	−	

We use the emmeans package on each LMM to extract the estimated marginal mean error for each method at each level of a selected factor or subset of factors. These marginal means are estimated by averaging the estimated conditional mean errors over the levels of all other factors using equal weights. We then use comparison contrasts that represent the difference between each of the marginal means and the overall marginal mean for each combination of Censoring and SampleSize. The figures in Sections 4.2.1 and 4.2.2 represent 95 % simultaneous confidence intervals for these differences. Thus, confidence intervals that are fully below or above 0 represent statistically significant differences from the mean (better and worse performances, respectively) among methods.

The confidence interval plots for the pilot study are available in the Supplementary Information.

#### 4.2.1 Point prediction

##### 4.2.1.1 10 % censoring

**Figure 2:**
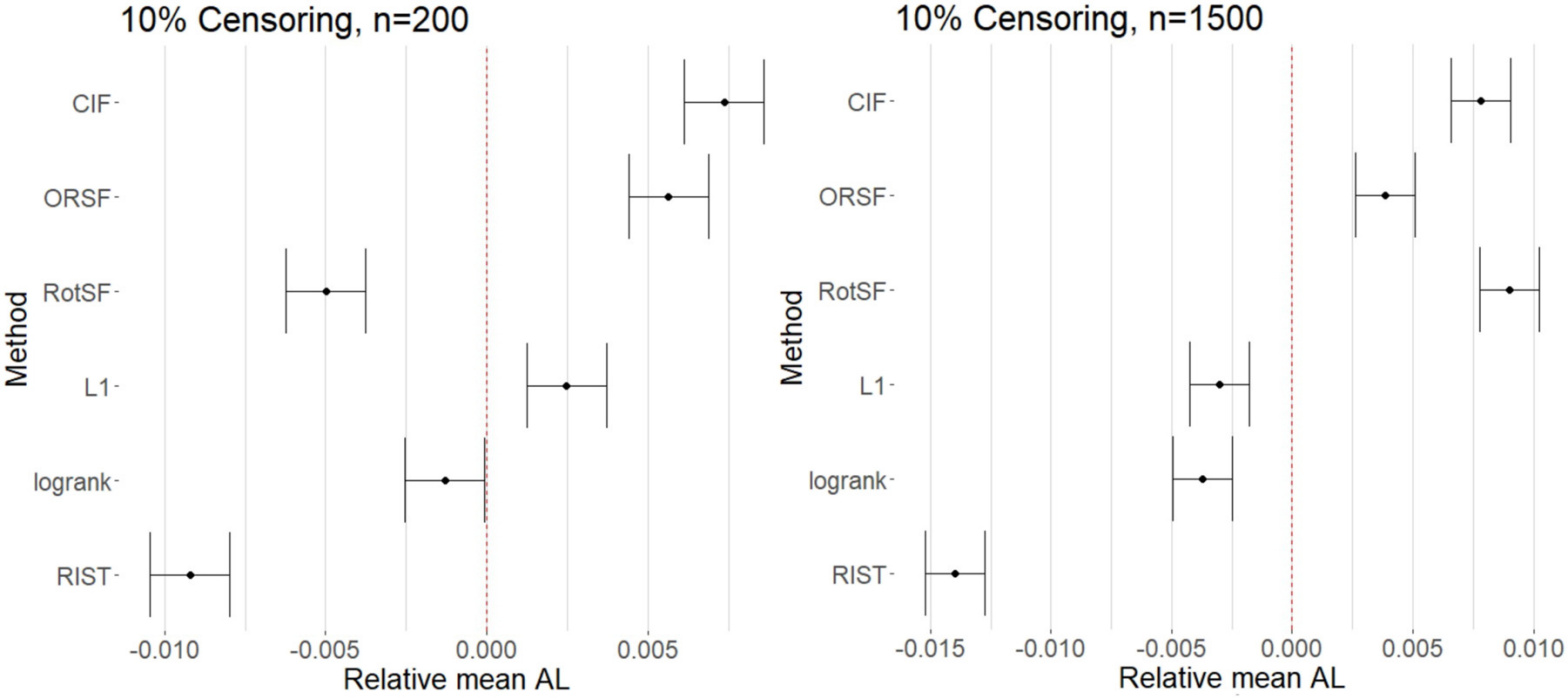
95 % simultaneous CIs for relative mean absolute loss (AL) for all methods at SampleSize = 200 (left) and SampleSize = 1500 (right) at Censoring = 10 %.

At our lowest Censoring level and at *n* = 200, RIST, RotSF, and RSF-log-rank had mean absolute losses significantly below the average across all methods, while CIF, ORSF, and *L*_1_ had mean absolute losses significantly above the average (see left panel of Figure 2). Moreover, we confirmed through pairwise comparisons that each method’s mean absolute loss was significantly different from all others (except for ORSF and CIF), so we can rank methods by performance for these factor levels, with RIST best and CIF worst.

At *n* = 1500 (see right panel of Figure 2), RIST, RSF-log-rank, and *L*_1_ had mean absolute losses significantly lower than the mean, while ORSF, CIF, and RotSF had mean absolute losses significantly higher than the mean. Once again, we confirmed through pairwise comparisons that RIST had mean absolute loss significantly lower than all other methods, while *L*_1_ and RSF-log-rank had mean absolute losses significantly lower than the three worse-than-average methods. Interestingly, RotSF performed significantly worse than average at this higher sample size (compared to better at the lower sample size). Conversely, *L*_1_ performed significantly better than average at the higher sample size and worse at the lower sample size.

##### 4.2.1.2 70 % censoring

**Figure 3:**
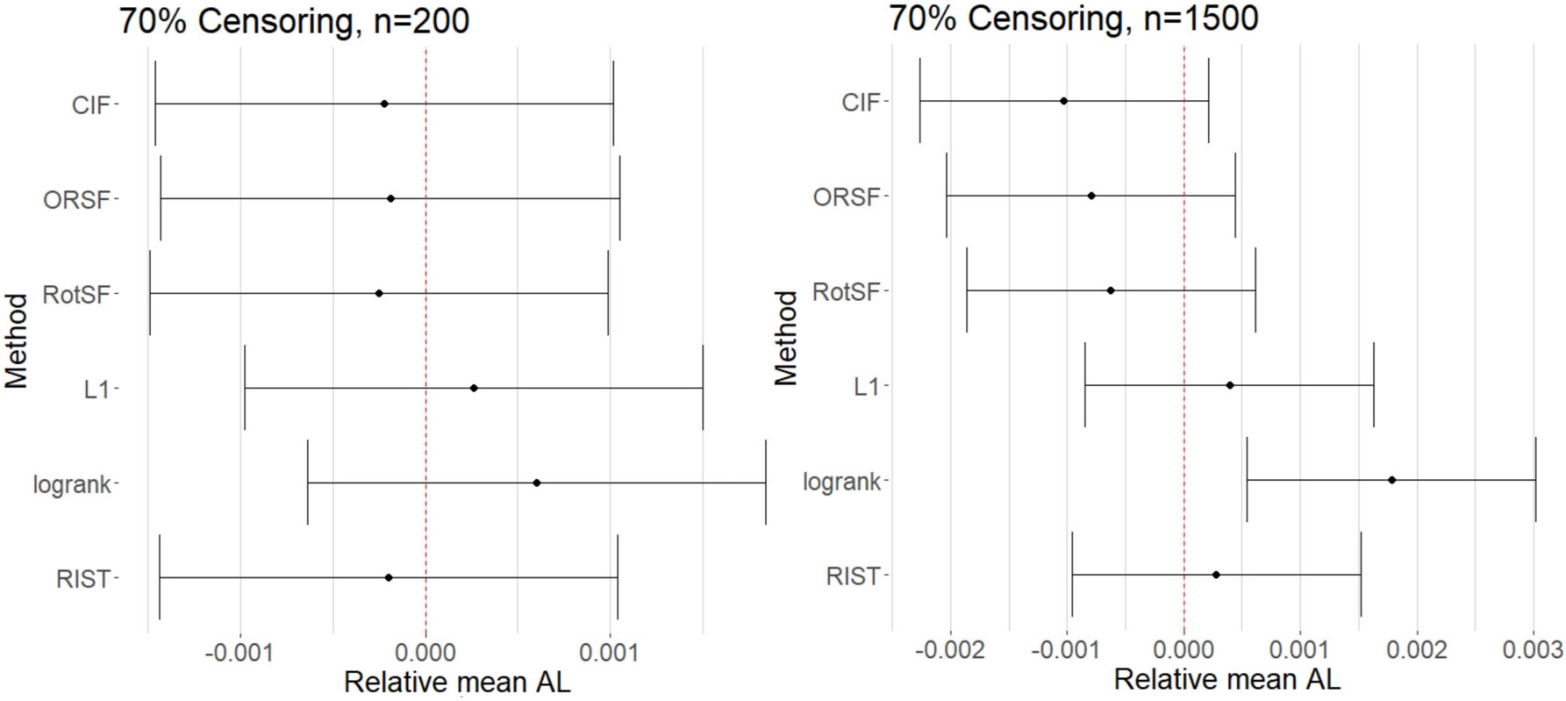
95 % simultaneous CIs for relative mean absolute loss (AL) for all methods at *n* = 200 (left) and *n* = 1500 (right) at 70 % censoring.

At our high Censoring level and at our smaller SampleSize level, comparisons indicated that no method had mean absolute loss that was significantly different from the mean across methods (Figure 3). These results are presumably due to the high variability in performance that is induced primarily by the high censoring rate and, to a lesser extent, the smaller sample size. Accordingly, at our higher SampleSize, no method had mean absolute loss significantly lower than the mean, while only RSF-log-rank performed significantly worse than average. The relative performance differences we identified at the 10 % Censoring level were almost entirely absent at 70 % Censoring. Based on our results, when Censoring is high, SampleSize is large, and the levels of other factors hard to determine, we recommend any of the methods except RSF-log-rank.

##### 4.2.1.3 C-index

Use of *E* = 1 − *C* as the error metric results in a substantially different picture of the relative performance of the methods. In particular, some methods that performed better than average according to C-index (i.e., CIF, ORSF) performed worse-than-average according to absolute loss, and vice versa (i.e., *L*_1_, RSF-log-rank). See confidence interval plots in Appendix B.

#### 4.2.2 Survival function estimation

The best- and worst-performing methods differed somewhat for survival function estimation compared to point prediction. Of the five methods compared, all but RSF-log-rank had some combinations of Censoring and SampleSize where their performance was better than average and some combinations where their performance was worse than average.

##### 4.2.2.1 10 % censoring

**Figure 4:**
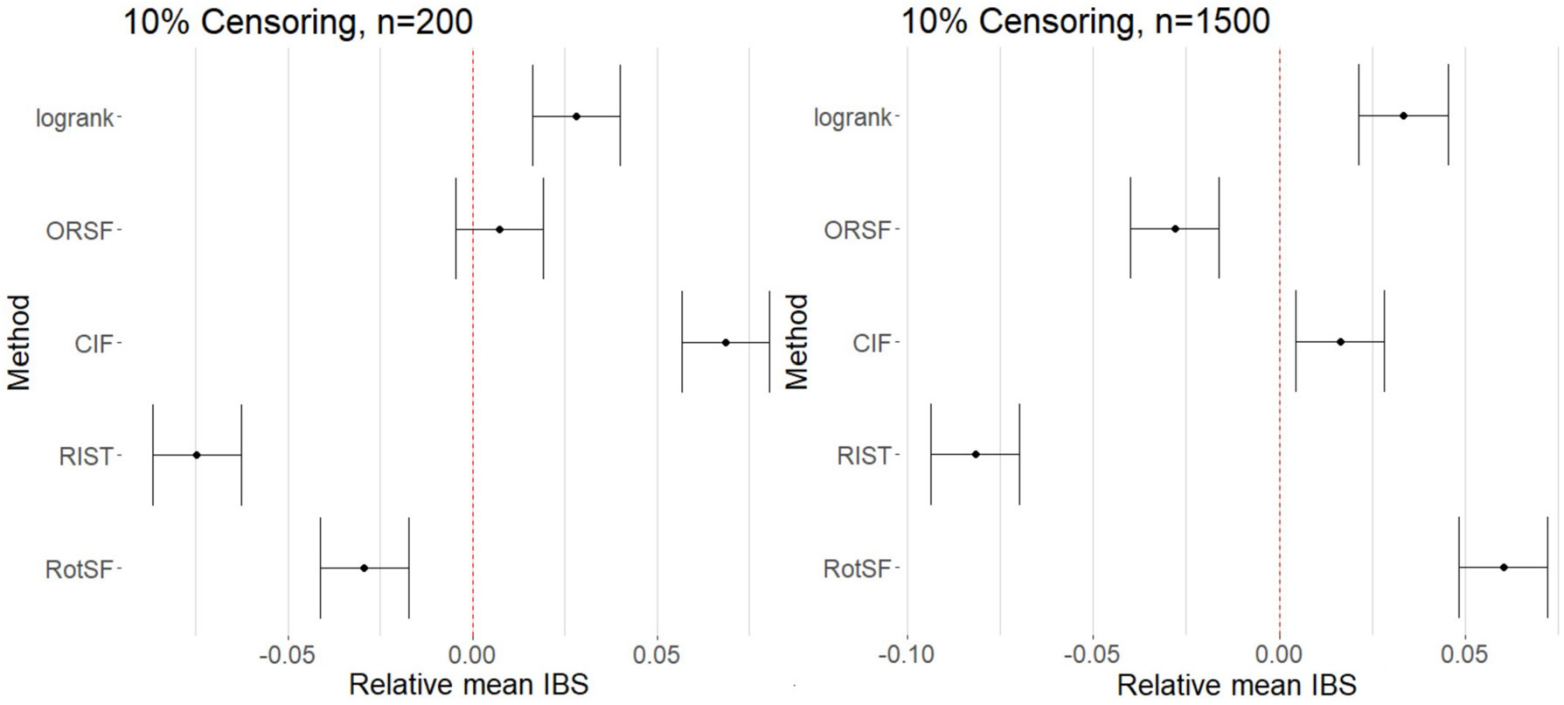
95 % simultaneous CIs for relative mean IBS for all methods at *n* = 200 (left) and *n* = 1500 (right) at 10 % censoring.

At our lowest Censoring level and at *n* = 200, both RIST and RotSF had mean IBS significantly lower than the mean across methods, while CIF and RSF-log-rank had significantly higher mean IBS, and ORSF had an indistinguishably different mean IBS (see left panel of Figure 4). As verified through pairwise comparisons, RIST performed significantly better than RotSF, and RSF-log-rank performed significantly better than CIF.

At *n* = 1500 (see right panel of Figure 4), RIST once again performed significantly better than average and significantly better than all other methods. Like at *n* = 200, CIF and RSF-log-rank had mean IBS significantly higher than the mean. Unlike at *n* = 200, RotSF had mean IBS significantly higher than the mean (and higher than that of CIF and RSF-log-rank, as verified through pairwise comparisons). As when absolute loss was used as the response, the lower Censoring level seems to lead to a reversal of the relative performance of RotSF at the two different sample sizes: RotSF appeared to struggle in a relative sense at the higher level of SampleSize when Censoring was at the low level.

##### 4.2.2.2 70 % censoring

**Figure 5:**
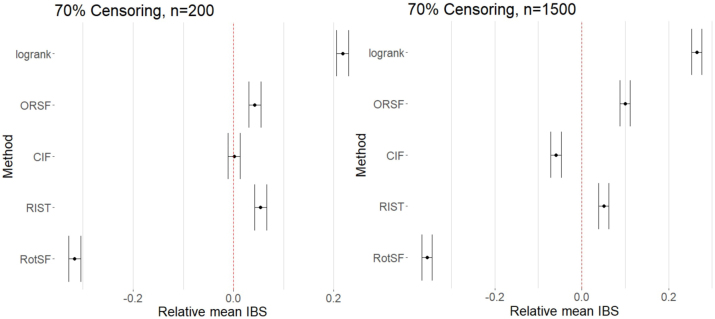
95 % simultaneous CIs for relative mean IBS for all methods at *n* = 200 (left) and *n* = 1500 (right) at 70 % censoring.

At our high Censoring level and at our smaller SampleSize, only RotSF had mean IBS significantly lower than average, while RSF-log-rank, ORSF, and RIST each had mean IBS significantly higher than the mean, and CIF performed indistinguishably differently from the mean (Figure 5). As confirmed through pairwise comparisons, RSF-log-rank performed much worse than all other methods.

At our higher SampleSize, RotSF yet again performed by far the best, achieving a mean IBS significantly lower than all other methods. Both RotSF and CIF performed better than the mean. The other three methods performed significantly worse than the mean, with RSF-log-rank performing by far the worst. Unlike with other high SampleSize FLCs, RotSF performed relatively *better* at these factor levels.

### 4.3 Recommendations for each sample size and censoring combination

Table 3 summarizes the better- and worse-than-average performing methods for each combination of Censoring and SampleSize for each error metric. Some clear trends are apparent, which we summarize with the following recommendations.

Point prediction:

1. Use RIST or RSF-log-rank when the censoring rate is low.
2. For smaller sample sizes, use RIST, RotSF, or RSF-log-rank.
3. Avoid CIF and ORSF, at least at lower censoring rates.
4. At higher censoring rates, avoid RSF-log-rank, but be aware that no method performs especially well.
5. Avoid RSF-log-rank-score, CRF-GRF, CRF-QRF, RSF-Brier, and RSF-C-index.
6. Use RIST-1 rather than RIST-*k* (*k* > 1) to save computational time.

Survival function estimation:

1. When both the sample size is small and censoring rate is low, use RotSF or RIST.
2. Avoid RSF-log-rank in any setting.
3. Use RotSF at higher censoring rates, and avoid RIST at higher censoring rates.
4. Avoid CIF at lower sample sizes.
5. Avoid RSF-log-rank-score, CRF-GRF, CRF-QRF, RSF-Brier, and RSF-C-index.
6. Use RIST-1 rather than RIST-*k* (*k* > 1) to save computational time.

We caution against overgeneralizing our results because it is unclear how representative our sample is of the broader population of survival data. Specifically, although we selected factor levels that arise commonly in survival datasets, their distribution in the population is presumably different from their distribution in our sample (recall that we used a balanced design).

Moreover, we acknowledge that some of the differences among methods that we report may be relatively small and that these differences may be overwhelmed by the noise in any given dataset. However, all our recommendations are based on observing differences among methods that were persistent for many FLCs.

We also recommend that practitioners be cautious about using C-index to choose a method of point prediction. As discussed in Section 4.1.2, the sample correlation between absolute loss and *E* = 1 − *C* is low, and the relative performance of methods according to C-index can look quite different from those according to absolute loss (which we view as the more informative metric). Therefore, treating *E* = 1 − *C* as a surrogate for absolute loss (which is not computable in practice) when choosing a method of point prediction may give misleading results. See the Supplementary Information for illustration and discussion of the observed relationship between these two error metrics.

### 4.4 Additional results: most influential factors and factor combinations

In this subsection, we discuss the most influential three-way interaction effects. We present these results separately because they concern at least one factor that is hard to determine and therefore are of more theoretical than practical interest. To identify these effects, we once again used the ANOVA output (see Appendix D) from each LMM, which indicates that the vast majority of effects are significant at the 5 % level. We also examined interaction plots to identify the more specific settings in which some methods are likely to perform better or worse than others, where “better performance” means a lower error.

Conveniently, the three-way interaction terms with the greatest F-values from the ANOVA ouptut align closely for all three error metrics. The magnitude of some interaction effects was often driven higher by a small subset of methods that performed much worse than the others, such that when we removed these weak methods from consideration, the estimated interaction effects were greatly reduced. In these cases, we discuss the relevant lower-order effects only.

#### 4.4.1 Point prediction

##### 4.4.1.1 DGM.Y:Censoring:Method

**Figure 6:**
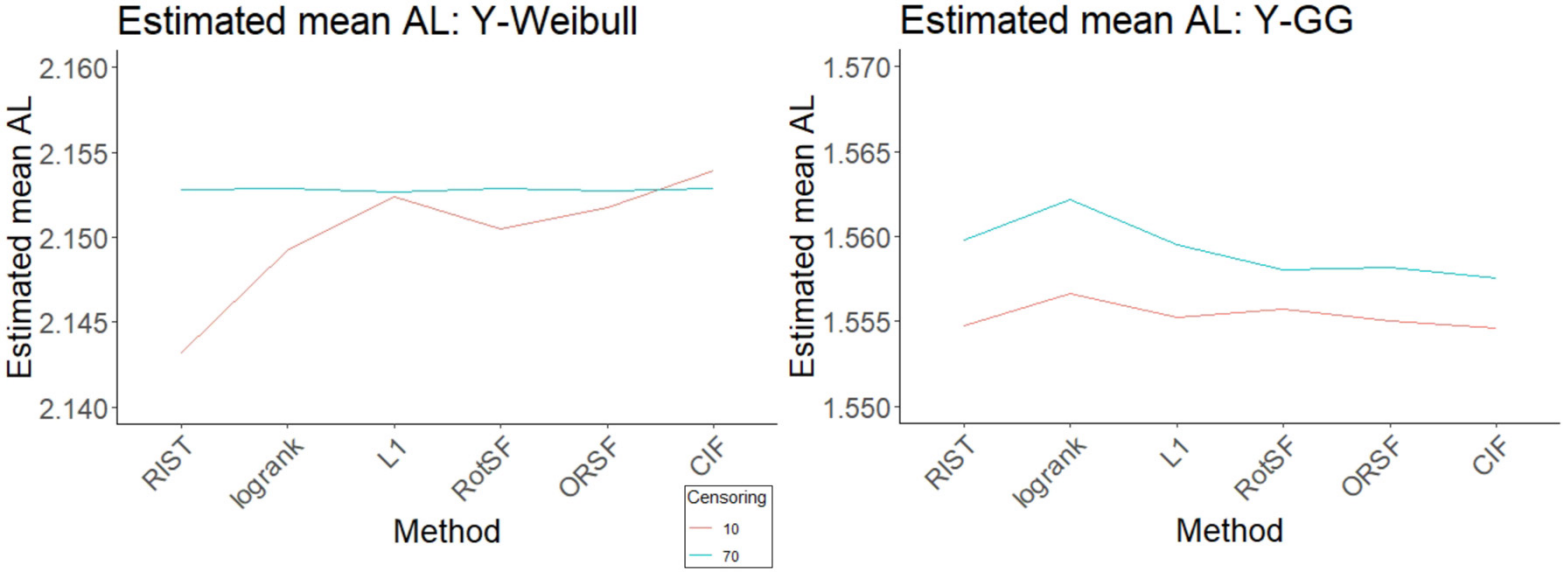
Estimated mean log(log(absolute loss)) interaction plot of Method:Censoring plotted for two levels of DGM-Y: Y-Weibull (left) and Y-GG (right).

Using absolute loss as the response, the most important three-way interaction effect according to the ANOVA table is DGM-Y:Censoring:Method. Interaction plots indicate the presence of this three-way effect (see Figure 6). Most methods appear to contribute to this effect, judging from how the two-way interactions for Y-Weibull look different from the two-interactions for Y-GG. (We omit Y-AFT as the results look similar to those for Y-Weibull.) Focusing just on Y-Weibull, the two-way interaction between Censoring and Method is obvious: at the 70 % Censoring level, no method performs discernibly better or worse than any other, whereas competitive advantages and disadvantages are very clear at the 10 % Censoring level. In contrast, focusing just on Y-GG, differences in performance among methods are much more consistent across Censoring levels.

##### 4.4.1.2 Censoring:SampleSize:Method

**Figure 7:**
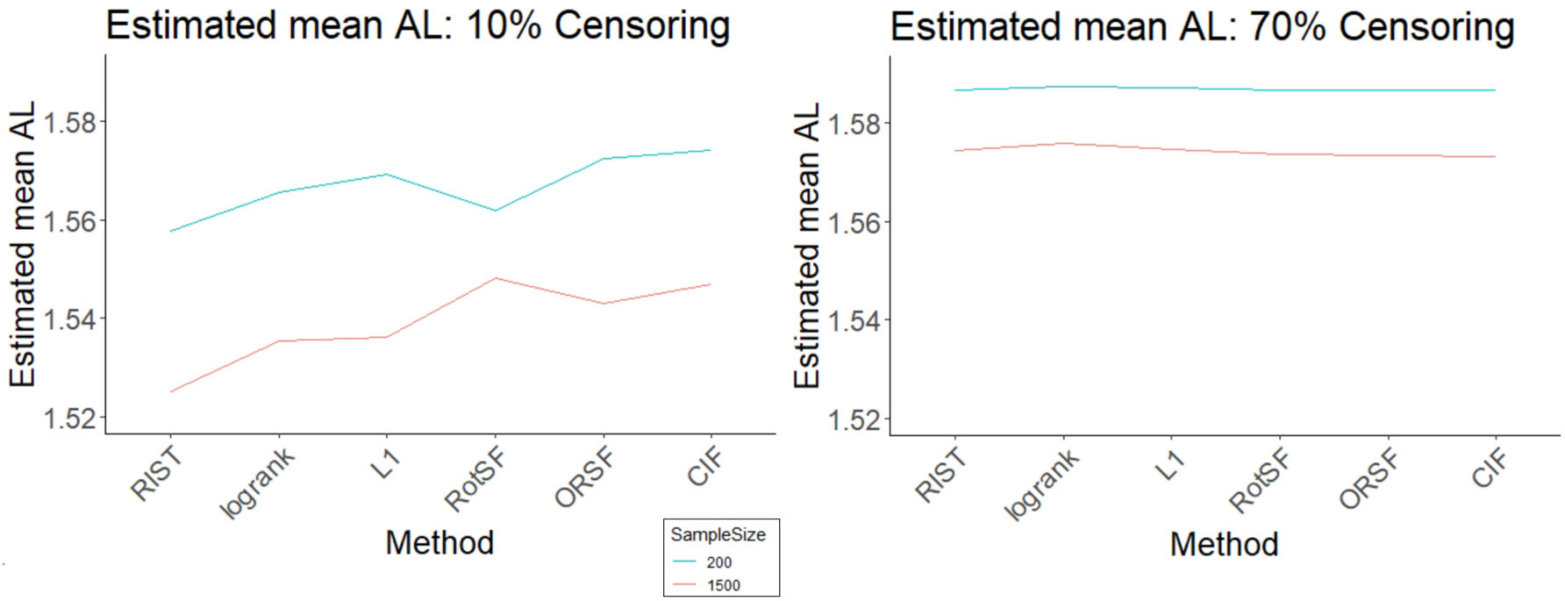
Estimated mean log(log(absolute loss)) interaction plot of Method:SampleSize plotted for Censoring = 10 % (left) and Censoring = 70 % (right).

**Figure 8:**
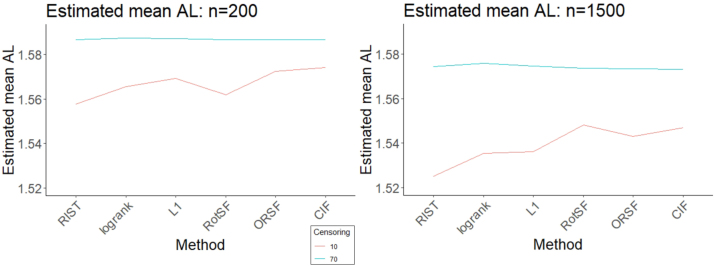
Estimated mean log(log(absolute loss)) interaction plot of Method:Censoring plotted for SampleSize = 200 (left) and Sample Size = 1500 (right).

Censoring:SampleSize:Method was the second most influential three-way interaction effect on absolute loss. Figures 7 and 8 suggest that the magnitude of this interaction is largely due to RotSF. This finding is consistent with the CI plots in Section 4.1.1, which revealed a reversal in RotSF’s relative performance at our two levels of SampleSize when holding Censoring constant. We refit the associated LMM without this method and observed that the F-values decreased substantially. These results confirm our recommendations in Section 4.3 to avoid RotSF for point prediction when both the sample size is large and censoring is low.

#### 4.4.2 Survival function estimation

##### 4.4.2.1 Censoring:Method:DGM-Y

**Figure 9:**
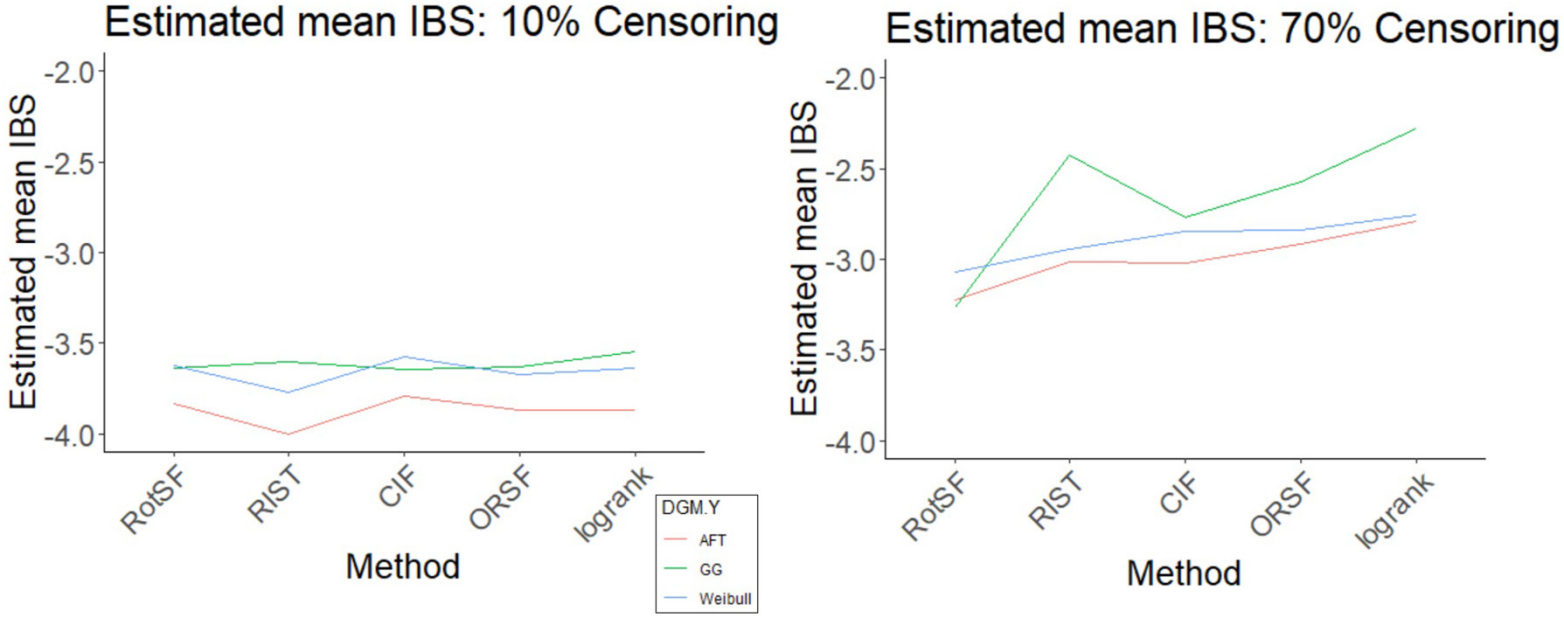
Estimated mean log(IBS) interaction plot of Censoring:Method plotted for Censoring = 10 % (left) and Censoring = 70 % (right).

**Figure 10:**
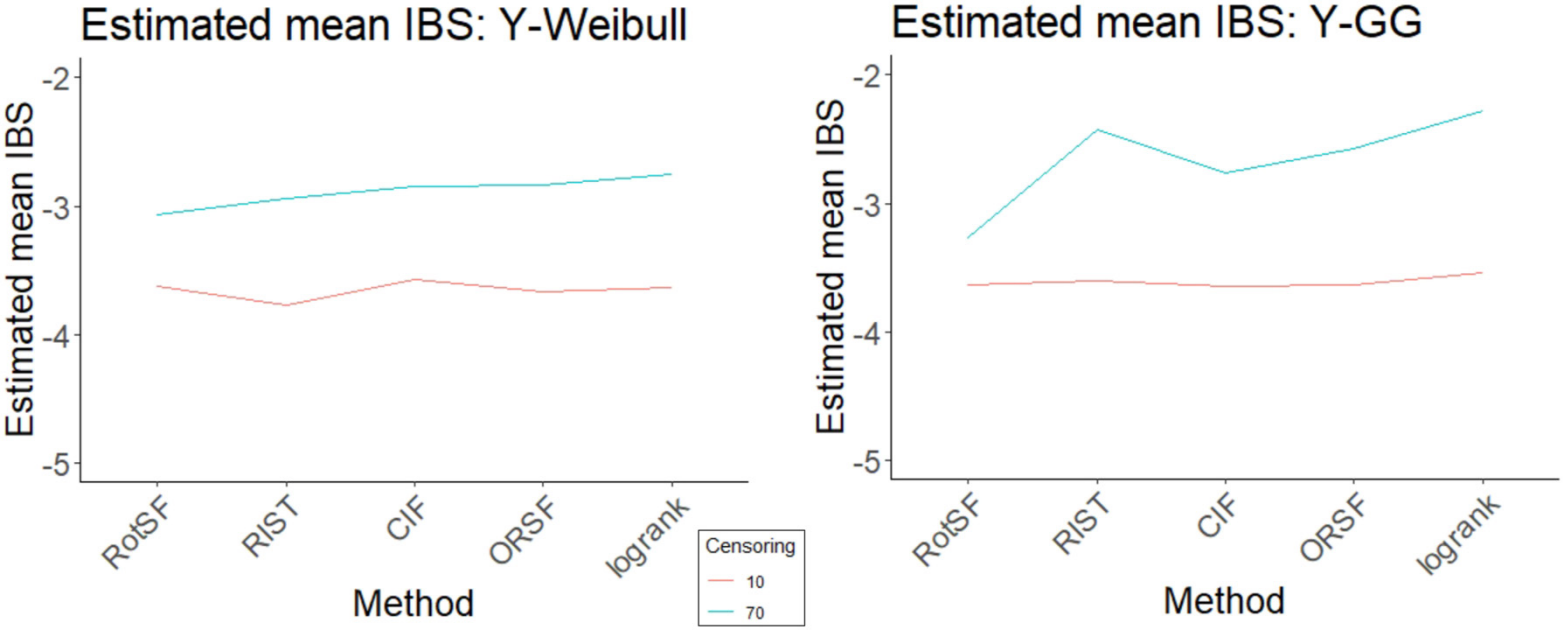
Estimated mean log(IBS) interaction plot of DGM-Y:Method plotted for Censoring 10 % (left) and Censoring 70 % (right).

When we used IBS as the response, the most important three-way interaction term was Censoring:Method:DGM-Y. The pairs of interaction plots in Figures 9 and 10 show visual evidence of three-way interactions. In particular, the DGM-Y:Method plots at two different Censoring levels suggest a three-way interaction driven primarily by methods’ different performance with Y-GG compared to that with other DGM-Y levels. In particular, compared to their performance for other DGM-Y levels (a) RotSF and CIF have comparatively lower IBS for Y-GG than other methods; (b) RIST and RSF-log-rank have relatively higher IBS; and (c) the average difference in response to Y-GG across all methods is bigger for the higher censoring level. These results underscore that, for survival function estimation, RIST’s relative struggles at our high Censoring level occurred primarily in the Y-GG setting. Moreover, the results exemplify how RotSF’s relative strength at our high Censoring level was consistent across levels of DGM-Y.

##### 4.4.2.2 SampleSize:Method:DGM-X

**Figure 11:**
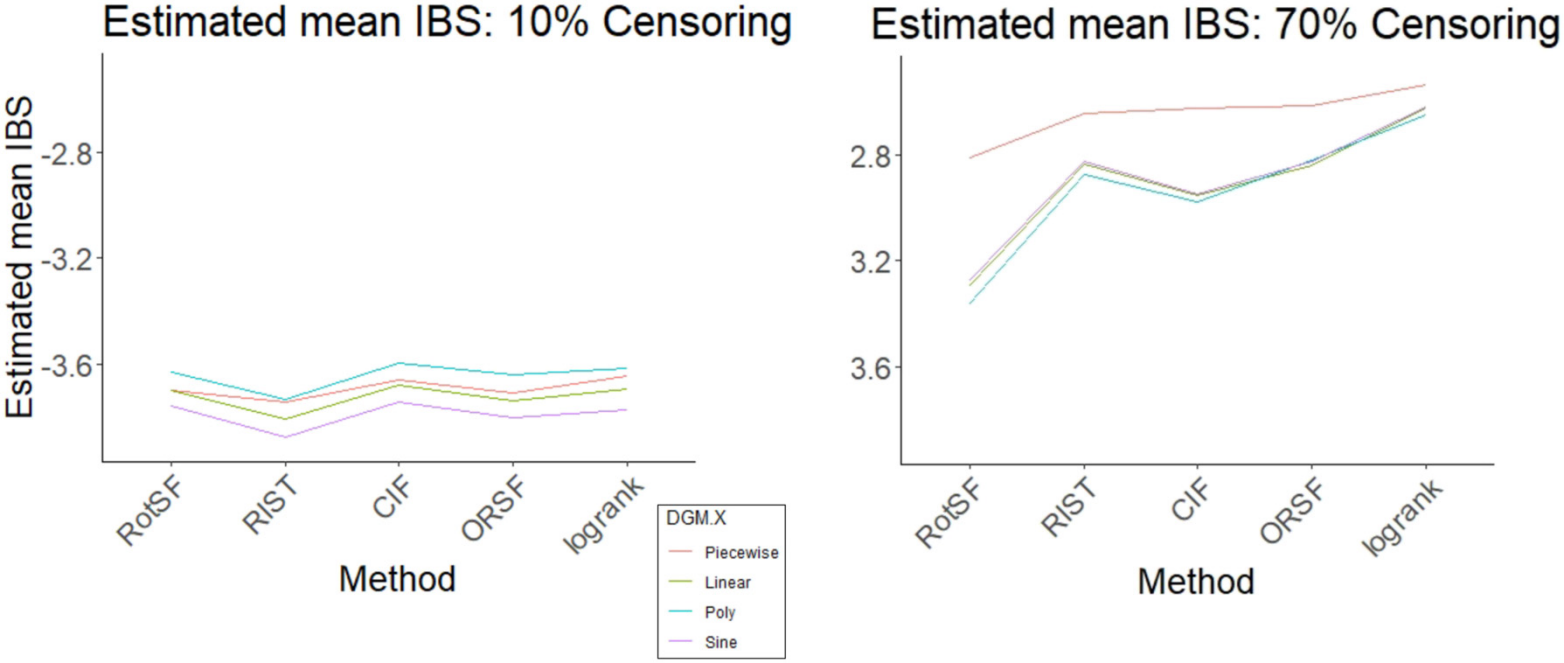
Estimated mean log(IBS) interaction plot of Method:DGM.X plotted for Censoring = 10 % (left) and Censoring = 70 % (right).

**Figure 12:**
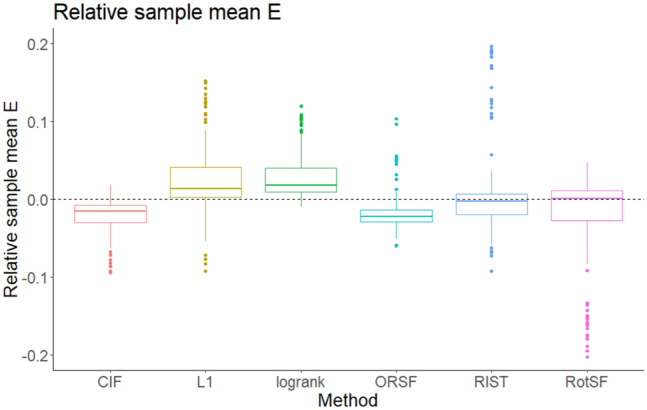
Relative sample mean E for all methods.

The next most important three-way interaction term according to the ANOVA table was SampleSize:Method:DGM-X. The patterns of differences in method performance change across each level of Censoring (see Figure 11). The bulk of the three-way interaction effect appears to be driven primarily by X-piecewise, the DGM-X level whose parameter values change depending on the values of *X*. Specifically, at the 10 % Censoring level, differences in method performance within each level of DGM-X are small and relatively consistent, whereas at the 70 % Censoring level, differences are larger, especially for X-piecewise.

## 5 Discussion

Our results raise the obvious question: *why* do some methods perform relatively better and worse than other methods in certain settings? In this section, we hypothesize that certain algorithmic differences in forest construction methods may explain their empirical performances in our simulation study.

The most common splitting criterion, RSF-log-rank, attained a middling or relatively good performance for point prediction at our low Censoring level, especially at our higher SampleSize level and when hazards did not cross. However, RSF-log-rank struggled with survival function estimation for other combinations of DGM-Y, Censoring, and SampleSize (see Table 8 in Appendix E). Recall that Y-Weibull has proportional hazards; Y-AFT has non-proportional, non-crossing hazards and *can* have crossing survival functions; and Y-GG has both crossing survival and crossing hazard functions. The log-rank test may have low power if the hazard functions are not proportional, so RSF-log-rank may lose power to detect survival function differences outside this setting, as was suggested by Moradian et al. [10]. For survival function estimation, RSF-log-rank did perform consistently below average for Y-GG (where hazard functions may cross). However, for point prediction, RSF-log-rank sometimes performed above average for Y-AFT. Moreover, for survival function estimation, even for Y-Weibull, where hazard functions do not cross, RSF-log-rank tended to perform below average. This latter finding suggests that some other methods (notably RotSF and RIST) have competitive advantages due to reasons unrelated to the power of the log-rank test.

In light of concerns about the performance of RSF-log-rank when hazard or survival functions cross, Moradian et al. [10] proposed *L*_1_ as an alternative splitting criterion. But our results showed that, for point prediction, *L*_1_ did not out-perform RSF-log-rank at any DGM-Y level for any combination of Censoring and SampleSize, even those where the hazard or survival functions cross. An explanation may be as follows. Like all the forest methods we are considering, *L*_1_ determines an optimal split of each node by assuming that the observations within each daughter node are identically distributed. However, the observations are likely far from identically distributed at nodes closer to the tops of trees. In such settings, the performance of the test based on *L*_1_ relative to that of the log rank test is unknown. Moreover, even if the hazard or survival functions cross for certain values of the covariates, the estimated hazard or survival functions for a given pair of daughter nodes may not cross. In other words, splitting criteria that are based on tests designed to handle crossing hazard or survival functions may not be necessary for identifying optimal splits in some locations within a tree. In addition, *L*_1_’s overall middling or subpar performance for Y-AFT and Y-GG may reflect strengths of other methods unrelated to the splitting criterion.

Referring back to Table 3, CIF performed worse than average at our low Censoring level using both error metrics at both levels of SampleSize. A major difference in the CIF algorithm compared to RSF-log-rank is that splitting terminates earlier if none of the candidate variables is significantly associated with the survival outcome. Earlier termination leads to shallower tree depth, which may lead to greater bias in both point predictors and survival function estimators. The effects of early termination may be more prominent when censoring is low because, in this case, the other methods may be able to form substantially larger and less biased trees using the large proportion of observed survival times.

In contrast, RIST performed better than average at our low Censoring level at both levels of SampleSize for point prediction and at our lower SampleSize level for survival function estimation. Recall that RIST recursively imputes censored observations using information from their estimated conditional survival distributions. One advantage of this procedure is that deeper trees can be grown due to a greater number of available survival times by, in effect, converting some censoring times into survival times (recall that terminal nodes generally must have a minimum of one survival time). In this way, the bias of the estimated quantities of interest may be reduced. At our low Censoring level, this imputation procedure does indeed appear to extract useful additional information from the data. At our high Censoring level, RIST either had no competitive advantage (using absolute loss) or performed worse than average (using IBS), suggesting that the imputed times may increase the bias or variance of the estimated survival functions. However, a closer inspection of the FLCs where RIST performed relatively poorly reveals that *all* were associated with Y-GG and that, for survival function estimation, RIST generally performed *better* than average at our high Censoring level for Y-Weibull and Y-AFT. So crossing hazards appear to pose a greater challenge to RIST than do high censoring rates, and the likely benefit of greater tree depth does not compensate for the challenges posed by Y-GG.

RotSF was unique in that it tended to perform better than average at our low SampleSize level and worse than average at our high SampleSize level (while still performing better in an absolute sense at the latter). For survival function estimation, RotSF also performed relatively better than all but one other method at our high Censoring level. That is, when information is more plentiful – i.e., a low censoring rate and a larger sample size – RotSF appears to do relatively worse, but with information scarcity, its algorithm appears to harness that information more efficiently. One possible explanation for these findings is as follows. RotSF begins by finding principal components of random subsets of the covariates. If the number of influential variables is sufficiently large, then their relationship with the response may be complex enough that it is best modelled with splits based on linear combinations of variables (e.g., principal components) rather than the variables themselves. In these cases, the most important principal components will explain more variance in the response than do the most important covariates, thus allowing for the construction of forests with better predictive performance. In an information-scarce setting (small sample size, high censoring rate), this procedure may thus outcompete other methods that randomly select covariates. But this advantage may be eliminated in more information-rich settings.

RotSF also performs comparably better in the presence of added noisy variables. In this setting, principal component analysis spreads the important variables within each subset into all the principal components in that subset. Thus, compared to other methods, fewer splits based entirely on noisy variables may occur, resulting in more accurate predictions.

While ORSF also transforms the covariates when building trees, it does so by fitting regularized Cox proportional hazards models to the data in each non-terminal node using random subsets of covariates and three different values of one of the regularization parameters. The estimated hazard functions (which are transformations of the subset of covariates chosen at that node) for each choice of regularization parameter become the candidate splitting variables. This model-based approach to building trees is quite different from the usual, entirely non-parametric approach of standard random forests. Perhaps due to these imposed parametric constraints, ORSF did not perform as well as RotSF – including in most settings at which RotSF performed above average. Even in the Y-Weibull setting, where the proportional hazards assumption is met, ORSF did not perform relatively better than other methods. The reason may be that, at each non-terminal node, not all important variables are included in the model, i.e., the model is misspecified.

## 6 Conclusions

We conducted an extensive review of all recent survival forest methods and a comprehensive simulation study to investigate the performance of some of these methods under various conditions. Based on the results from this study, we summarized relative performance differences in a variety of settings and determined recommendations for the practitioner.

Most methods we retained for the comprehensive simulation study (CIF, RSF-log-rank, RIST-1, RotSF, *L*_1_, and ORSF) were competitive for point prediction under different settings, so we would not *a priori* rule out any of these methods for this purpose. For survival function estimation, RSF-log-rank tended to perform consistently below average; other methods were competitive overall, while excelling or struggling in more specific settings.

In contrast, we ruled out RSF-log-rank-score, RSF-C-index, RSF-Brier-score, CRF-GRF, and CRF-QRF, which we tested in the pilot study, due to their more widespread subpar performance. In particular, we found that RSF-log-rank-score was by far the worst-performing method for both point prediction and survival function estimation. Moreover, we removed RIST-3 from the comprehensive study because we found that it did not perform substantially better than RIST-1 in the pilot study but takes considerably more computational time.

Among other notable results, for point prediction, when censoring was low, RotSF performed better than average at our lower sample size and worse than average at our higher sample size. Moreover, when censoring was low, both RIST-1 and RSF-log-rank performed better than average, except at Y-GG (where they performed no differently from average). Interestingly, *L*_1_ had no advantage over RSF-log-rank for any combination of censoring and sample size levels. CIF and ORSF both performed below average when censoring was low. At our high censoring level, no method performed consistently better than average.

For survival function estimation, perhaps the most notable result was the relative performance advantage of RotSF in information-scarce settings (low sample size and/or high censoring rate) and relative performance *dis*advantage in information-rich settings (high sample size and low censoring). RIST-1 performed better than average when censoring was low, except at Y-GG. RSF-log-rank performed consistently below average for every combination of censoring and sample size. Whereas ORSF consistently struggled when censoring was high, CIF consistently struggled when censoring was low.

Plenty of opportunities exist for future work. Testing our hypotheses about the reasons for methods’ relative performance advantages and disadvantages is of particular importance. For example, is tree depth a key factor in determining the below-average performance of CIF and above-average performance of RIST in certain settings?

Another factor that could be investigated for its effect on method performance is the signal-to-noise ratio (SNR). We used a constant, relatively high SNR, but relative performance of the methods might differ under different SNR settings. For example, Mentch and Zhou [36] found that, to minimize error, low SNR settings are best accompanied by a smaller number of candidate variables from which to select an optimal split.

We also used the default tuning parameter values of each forest method. Our results might have differed had we tuned each method on each dataset.

Perhaps most importantly for practitioners, an implementation of these methods via packages on CRAN would be tremendously beneficial, especially if one package could amalgamate all methods. (We remind the reader that we obtained code for RIST and *L*_1_ from the authors who proposed them and the code for RotSF and CRF from their authors’ GitHub pages.) This synthesis would simplify the comparison of the performance of the methods by standardizing the choice of point predictions and error metrics across methods. Without this standardization, inconsistencies across methods can occur. For instance, we used the estimated median as the point prediction when computing the C-index, whereas Ishwaran et al. [6] used “predicted mortality” in their randomForestSRC package. Similarly, the range of the estimated survival function for a given set of covariates differs across packages. Like Kaplan-Meier and other non-parametric methods, most survival forest methods provide probability estimates at each observed survival time in the training set. However, the *L*_1_ implementation, for example, provides estimates of the survival function only at certain subsets of the observed survival times.

Another challenge worthy of investigation is the issue of point prediction (via the estimated median) when the estimated survival function does not drop below 0.5.

We caution that the datasets that we generated to evaluate method performance should not be misconstrued as a representative sample of all survival datasets. For example, one-third of all FLCs were at Y-GG, but crossing hazards may not be a property seen with such a high frequency in practice. Rather, we sought to investigate a range of possible factors, factor levels, and FLCs that a practitioner might encounter and to understand how methods might interact with them. Moreover, we did not test the methods on real datasets because the number of publicly available survival datasets is very limited, particularly those with a substantial number of covariates. We were unable to locate a repository of such datasets, but it would be a welcome addition to the resources available to researchers in machine learning.

We also recommend investigating method performance at more censoring levels to inform us when some methods – especially those that performed very differently at our 10 % and 70 % censoring levels – start to suffer or excel. Relatedly, do methods that require estimation of the censoring distribution perform relatively poorly with *lower* censoring rates?

In summary, we have reviewed and compared all recent survival forest methods proposed in the literature and discovered various settings in which some perform relatively better or worse. Our results provide guidance to practitioners about which methods to use for their specific application. Our work also paves the way for future research on the mechanisms underlying these methods and the development of new methods with even better performance.

## Supplementary Material

Supplementary Material Details

## Figures and Tables

**Figure 13:**
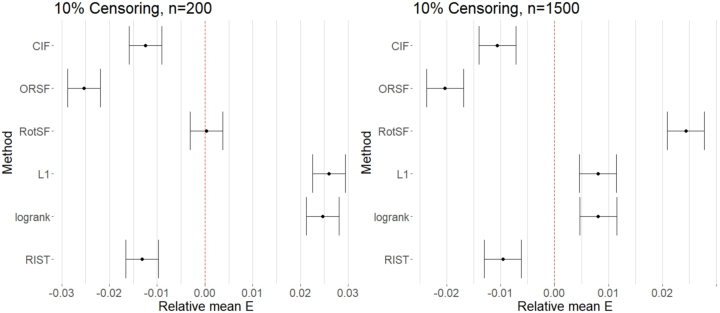
95 % simultaneous CIs for relative mean *E* = 1 − *C* for all methods at SampleSize = 200 (left) and SampleSize = 1500 (right) at Censoring = 10 %.

**Figure 14:**
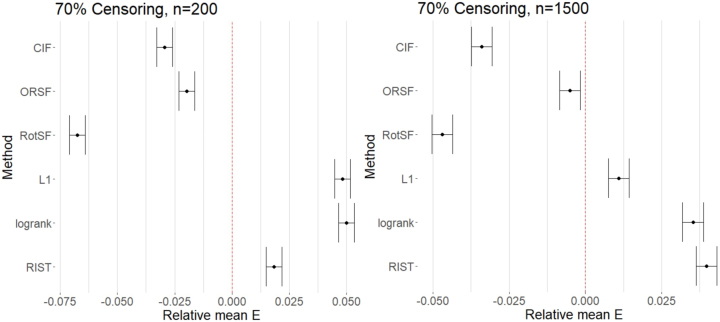
95 % simultaneous CIs for relative mean *E* = 1 − *C* for all methods at SampleSize = 200 (left) and SampleSize = 1500 (right) at Censoring = 70 %.

## Appendix A: Pseudo *R* ^2^ and signal-to-noise ratio

In order to enforce consistent signal-to-noise ratios (SNRs) across factor-level combinations, we used a pseudo *R* ^2^ that is a function of the conditional means and variances associated with survival times within each DGM. The conditional mean and variance associated with the Weibull, lognormal, and generalized gamma DGM-Ys are displayed in Table 4.

**Table 4:** Conditional mean and variance associated with the Weibull, lognormal, and generalized gamma DGM-Ys.

Model	Weibull	Log-normal	Generalized gamma
*E*(*T*|*X* = *x*)	$\frac{\Gamma \left(1+1/\rho \right)}{{\left(\lambda \exp\left\{x^{\prime }\beta \right\}\right)}^{1/\rho }}$	$\exp\left(x^{\prime }\beta +\frac{\sigma^{2}}{2}\right)$	$\frac{a\cdot \exp\left(x^{\prime }\beta \right)\Gamma \left(\left\{bk+1\right\}/b\right)}{\Gamma \left(k\right)}$
Var(*T*|*X* = *x*)	$\frac{\Gamma \left(1+2/\rho \right)-{\left[\Gamma \left(1+1/\rho \right)\right]}^{2}}{{\left(\lambda \exp\left\{x^{\prime }\beta \right\}\right)}^{2/\rho }}$	(exp(*σ* ^2^) − 1) exp(2**x′*β*** + *σ* ^2^)	$a\cdot \exp\left(x^{\prime }\beta \right)\left(\frac{\Gamma \left(\left\{bk+2\right\}/b\right)}{\Gamma \left(k\right)}-\frac{{\left[\Gamma \left(\left\{bk+1\right\}/b\right)\right]}^{2}}{\Gamma \left(k\right)}\right)$

We defined the pseudo *R* ^2^ for Y-Weibull, for example, as

$$R_{\text{W}\;\text{eibull}}^{2}=\frac{\mathrm{Var}\left[\frac{\Gamma \left(1+1/\rho \right)}{{\left(\lambda \exp\left\{X^{\prime }\beta \right\}\right)}^{1/\rho }}\right]}{\mathrm{Var}\left[\frac{\Gamma \left(1+1/\rho \right)}{{\left(\lambda \exp\left\{X^{\prime }\beta \right\}\right)}^{1/\rho }}\right]+E\left[\frac{\Gamma \left(1+2/\rho \right)-{\left[\Gamma \left(1+1/\rho \right)\right]}^{2}}{{\left(\lambda \exp\left\{X^{\prime }\beta \right\}\right)}^{2/\rho }}\right]}.$$

The SNR was thus

$${\text{SNR}}_{\text{W}\;\text{eibull}}=\frac{R_{\text{W}\;\text{eibull}}^{2}}{1-R_{\text{W}\;\text{eibull}}^{2}}.$$

The moments contained in $R_{\text{W}\;\text{eibull}}^{2}$ are complex and depend on the distribution of **X**. We therefore approximate them with their empirical moments (obtained by simulating 1500 values of **X** for each level of DGM-Y and DGM-X). We defined the pseudo *R* ^2^ and SNR for the other DGM-Ys analogously.

## Appendix B: Plots of *E* = 1 − *C*

The boxplots for the *E* = 1 − *C* error metric by Method (analogous to the boxplots in Section 4.1) are displayed in Figure 12.

When we used *E* = 1 − *C* to measure error, ORSF and CIF had lower quartiles entirely below the mean, while *L*_1_ and RSF-log-rank had upper quartiles entirely above the mean. RIST and RotSF exhibited more varied performances.

In Section 4.2.1, we presented plots of simultaneous confidence intervals for the relative mean absolute loss values from our comprehensive study for each combination of Censoring and SampleSize. We present the analogous plots for *E* in Figures 13 and 14.

## Appendix C: Linear predictors used in DGM-X

Four of the ten covariates we used in our linear predictors are five-level categorical variables. We used indicator variables to denote these levels (other than the baseline levels) using *X*_7.1_, *X*_7.2_, …, *X*_10.4_.

X-baseline: *X*_1_ *β*_1_ + *X*_2_ *β*_2_ + ⋯*X*_10.4_ *β*_22_

X-poly-int: $X_{1}\beta_{1}+X_{2}^{2}\beta_{2}+X_{3}\beta_{3}+X_{4}^{2}\beta_{4}+X_{3}X_{4}\beta_{5}+X_{4}^{2}X_{5}\beta_{6}+X_{6}^{3}\beta_{7}+X_{7.1}\beta_{8}+\cdots +X_{10.4}\beta_{23}$

X-sine: *X*_1_ *β*_1_ + sin(*X*_2_)*β*_2_ + *X*_3_ *β*_3_ + ⋯ + *X*_10.4_ *β*_22_

X-piecewise: $X_{1}\beta_{1}+1_{X_{2}<0.3}X_{2}\beta_{2}+1_{0.3\leq X_{2}<0.8}X_{2}\beta_{3}+1_{X_{2}\geq 0.8}X_{2}\beta_{4}+1_{X_{3}<0.3}X_{3}\beta_{5}+1_{0.3\leq X_{3}<0.8}X_{3}\beta_{6}+1_{X_{3}\geq 0.8}X_{3}\beta_{7}+X_{4}\beta_{8}+\cdots +X_{10.4}\beta_{26}$

Note that the values of ***β*** varied across each DGM-Y and DGM-X.

## Appendix D: ANOVA output

Tables 5 [Table j_ijb-2023-0056_tab_006]–7 display the ANOVA tables for the comprehensive study using absolute loss, IBS, and the C-index as the response, respectively.

**Table 5:** ANOVA table (type III) for the comprehensive simulation study using absolute loss as the response.

Coef	numDF	denDF	*F*-value	*p*-value
(Intercept)	1	6995	2201.38	<0.0001
DGM.Y	2	1399	1487.50	<0.0001
DGM.X	3	1399	58.17	<0.0001
Censoring	1	1399	44.60	<0.0001
ExtraVars	2	1399	0.46	0.6318
SampleSize	1	1399	9.63	0.0020
Method	5	6995	135.89	<0.0001
DGM.Y:DGM.X	6	1399	1059.15	<0.0001
DGM.Y:Censoring	2	1399	27.68	<0.0001
DGM.Y:ExtraVars	4	1399	0.75	0.5595
DGM.Y:SampleSize	2	1399	2.68	0.0690
DGM.X:Censoring	3	1399	1.14	0.3311
DGM.X:ExtraVars	6	1399	1.65	0.1310
DGM.X:SampleSize	3	1399	0.11	0.9547
Censoring:ExtraVars	2	1399	0.65	0.5227
Censoring:SampleSize	1	1399	2.81	0.0939
ExtraVars:SampleSize	2	1399	0.93	0.3936
DGM.X:Method	15	6995	10.51	<0.0001
Censoring:Method	5	6995	123.52	<0.0001
ExtraVars:Method	10	6995	112.94	<0.0001
SampleSize:Method	5	6995	100.59	<0.0001
DGM.Y:Method	10	6995	54.19	<0.0001
DGM.X:Censoring:Method	15	6995	5.12	<0.0001
DGM.X:ExtraVars:Method	30	6995	1.80	0.0046
DGM.X:SampleSize:Method	15	6995	1.44	0.1201
Censoring:ExtraVars:Method	10	6995	49.92	<0.0001
Censoring:SampleSize:Method	5	6995	52.95	<0.0001
ExtraVars:SampleSize:Method	10	6995	14.92	<0.0001
DGM.Y:DGM.X:Method	30	6995	3.53	<0.0001
DGM.Y:Censoring:Method	10	6995	92.62	<0.0001
DGM.Y:ExtraVars:Method	20	6995	32.53	<0.0001
DGM.Y:SampleSize:Method	10	6995	38.16	<0.0001

**Table 6: j_ijb-2023-0056_tab_006:** ANOVA table (type III) for the comprehensive simulation study using IBS as the response.

Coef	numDF	denDF	*F*-value	*p*-value
(Intercept)	1	55,966,149.326	<0.0001	<0.0001
DGM.Y	2	1399	55.49	<0.0001
DGM.X	3	1399	4.53	0.0036
Censoring	1	1399	364.36	<0.0001
ExtraVars	2	1399	2.07	0.1271
SampleSize	1	1399	98.84	<0.0001
Method	4	5596	21.25	<0.0001
DGM.Y:DGM.X	6	1399	15.96	<0.0001
DGM.Y:Censoring	2	1399	17.31	<0.0001
DGM.Y:ExtraVars	4	1399	0.71	0.5868
DGM.Y:SampleSize	2	1399	6.63	0.0014
DGM.X:Censoring	3	1399	61.55	<0.0001
DGM.X:ExtraVars	6	1399	1.28	0.2651
DGM.X:SampleSize	3	1399	3.05	0.0278
Censoring:ExtraVars	2	1399	2.17	0.1147
Censoring:SampleSize	1	1399	3.04	0.0817
ExtraVars:SampleSize	2	1399	4.86	0.0079
DGM.X:Method	12	5596	11.64	<0.0001
Censoring:Method	4	5596	41.97	<0.0001
ExtraVars:Method	8	5596	61.59	<0.0001
SampleSize:Method	4	5596	83.74	<0.0001
DGM.Y:Method	8	5596	5.25	<0.0001
DGM.X:Censoring:Method	12	5596	72.91	<0.0001
DGM.X:ExtraVars:Method	24	5596	0.59	0.9452
DGM.X:SampleSize:Method	12	5596	2.87	0.0006
Censoring:ExtraVars:Method	8	5596	12.60	<0.0001
Censoring:SampleSize:Method	4	5596	62.61	<0.0001
ExtraVars:SampleSize:Method	8	5596	30.02	<0.0001
DGM.Y:DGM.X:Method	24	5596	39.63	<0.0001
DGM.Y:Censoring:Method	8	5596	176.62	<0.0001
DGM.Y:ExtraVars:Method	16	5596	44.72	<0.0001
DGM.Y:SampleSize:Method	8	5596	56.58	<0.0001

**Table 7:** ANOVA table (type III) for the comprehensive simulation study using C-index as the response.

Coef	numDF	denDF	*F*-value	*p*-value
(Intercept)	1	6995	340.28	<0.0001
DGM.Y	2	1399	322.93	<0.0001
DGM.X	3	1399	2.16	0.0910
Censoring	1	1399	3.42	0.0645
ExtraVars	2	1399	0.46	0.6302
SampleSize	1	1399	174.85	<0.0001
Method	5	6995	19.47	<0.0001
DGM.Y:DGM.X	6	1399	146.50	<0.0001
DGM.Y:Censoring	2	1399	603.05	<0.0001
DGM.Y:ExtraVars	4	1399	2.59	0.0350
DGM.Y:SampleSize	2	1399	133.23	<0.0001
DGM.X:Censoring	3	1399	0.31	0.8079
DGM.X:ExtraVars	6	1399	1.43	0.2007
DGM.X:SampleSize	3	1399	15.18	<0.0001
Censoring:ExtraVars	2	1399	1.07	0.3431
Censoring:SampleSize	1	1399	0.38	0.5373
ExtraVars:SampleSize	2	1399	0.56	0.5737
DGM.Y:Method	10	6995	37.30	<0.0001
DGM.X:Method	15	6995	5.63	<0.0001
Censoring:Method	5	6995	18.95	<0.0001
ExtraVars:Method	10	6995	19.54	<0.0001
SampleSize:Method	5	6995	38.16	<0.0001
DGM.Y:DGM.X:Method	30	6995	51.52	<0.0001
DGM.Y:Censoring:Method	10	6995	333.43	<0.0001
DGM.Y:ExtraVars:Method	20	6995	18.13	<0.0001
DGM.Y:SampleSize:Method	10	6995	34.29	<0.0001
DGM.X:Censoring:Method	15	6995	37.87	<0.0001
DGM.X:ExtraVars:Method	30	6995	1.10	0.3191
DGM.X:SampleSize:Method	15	6995	4.22	<0.0001
Censoring:ExtraVars:Method	10	6995	13.45	<0.0001
Censoring:SampleSize:Method	5	6995	20.72	<0.0001
ExtraVars:SampleSize:Method	10	6995	21.17	<0.0001

## Appendix E: Additional summary of results

Based on the results from our comprehensive study, Table 8 summarizes the better- and worse-than-average performing methods for each combination of DGM-Y, Censoring, and SampleSize for each error metric.

**Table 8:** The better-than-average (“+”) and worse-than-average (“−”) performing survival forest construction methods for each combination of DGM-Y, Censoring (*C*), and SampleSize (*n*) for each error metric.

Loss	DGM-Y	*C*	*n*	CIF	ORF	RIST-1	RotSF	RSF-log-rank	L1
AL	Weibull	10	200	–		+	+		–
			1500	–		+	–	+	
		70	200				–		
			1500				+		
	AFT	10	200	–	–	+	+	+	–
			1500	–	–	+	–	+	+
		70	200				+		–
			1500		+		–		
	GG	10	200				+		
			1500				–		
		70	200				–		
			1500	+			+	–	
IBS	Weibull	10	200	–		+	+	–	
			1500	–	+	+	–		
		70	200	–		+	+	–	
			1500	–	–	+	+	–	
	AFT	10	200	–	–	+	+		
			1500	–	+	+	–		
		70	200		–		+	–	
			1500	+	–	+	+	–	
	GG	10	200		+			–	
			1500	+		–	+	–	
		70	200	+	–	–	+	–	
			1500	+	–	–	+	–	
							

## Appendix F: Experimental design concepts and terminology

This appendix offers a more technical explanation of Section 3.4 using standard experimental design concepts and terminology as described in books on experimental design such as Wu and Hamada [37].

Our experiment follows a split-plot design, where the factors that affect the data simulation are whole-plot factors and Method is the subplot factor (because its levels are applied to each FLC). A complete factorial design in the whole plot therefore consists of 3 × 4 × 3 × 2 × 3 = 216 FLCs. In a split-plot design, treatment factors are assigned to units of different sizes, where subplot units are nested within whole-plot units. Whole-plot factors are assigned to larger units, whereas subplot factors are assigned to the smaller, nested units. Therefore, multiple measurements are made on each whole plot (generally one per subplot). In our experiment, we simulated datasets under combinations of the factors (i.e., the FLCs) described in Sections 3.1.2–3.1.5. On each dataset, we repeatedly observed the error metric (once per forest method). Thus, the FLCs are whole-plot factors, and the methods are subplot factors.

While a full factorial design uses all possible FLCs, a fractional factorial design uses only a subset of FLCs. The subset is chosen in a way that allows estimation of main effects and some interactions, while confounding, or “aliasing”, the effects of higher-order interactions with lower-order interactions. This design is particularly valuable for reducing the total number of runs when a full factorial would be impractical. Fractional factorials are justified using the effect hierarchy principle, which suggests that lower-order effects (including main effects) tend to be more pronounced than higher-order interaction effects [37]. In our study, this principle was invoked to prioritize the study of main effects and two-way interactions.

Fractional factorial designs are generally chosen to satisfy some optimality criterion relating to the variance of the estimators of the remaining effects in the experiment. Fractional designs have been published for various fractions of experiments with certain numbers of factors and levels, but not for all. To our knowledge, none is available for our experiment, which included 3 × 4 × 3 × 2 × 3 = 216 FLCs. The JMP statistical software has a custom design capacity that can be used to generate specialized designs [38]. In our study, JMP helped to identify a fractional, D-optimal design that would achieve our goals. As mentioned in Section 3.4, the result was a design with only 60 FLCs. We generated 10 datasets (replicates) per FLC and applied each method to each dataset.
