# Supplementary material for: Random forests for survival data: which methods work best and under what conditions?
Source: Int J Biostat. 2024 Apr 24;20(2):315–45. doi: 10.1515/ijb-2023-0056 (PMC11661562; doi:10.1515/ijb-2023-0056)
Supplement: Supplementary file 1 — Supplementary Material Details [file j_ijb-2023-0056_suppl_001.pdf]

Supplementary Information

S1. Comprehensive study - relationship between absolute loss and C-index

In Section 4.1.1, we reported on the observed relationship between absolute loss and $E = 1 - C$ for each method within FLC. We illustrate this relationship via histograms in Figure 1, which displays sample correlations of absolute loss and E for each of the six methods in our comprehensive study *within* FLC. Note that each sample correlation is based on only 10 pairs of absolute loss and E (and thus the correlations are likely noisy estimates of the true correlation). The low observed correlations suggest that we should not use E as a surrogate for absolute loss.

S2. Pilot study - boxplots

In Section 4.1, we presented boxplots of the relative sample mean error values from our comprehensive study. Here, we present the analogous boxplots for our pilot study, which include all 12 methods listed in Table 2.

S3. Pilot study - confidence interval plots

In Section 4.2, we presented plots of simultaneous confidence intervals for the relative mean error values from our comprehensive study for each combination of `Censoring` and `SampleSize`. Here, we present the analogous plots for our pilot study, which include all 12 methods listed in Table 2.

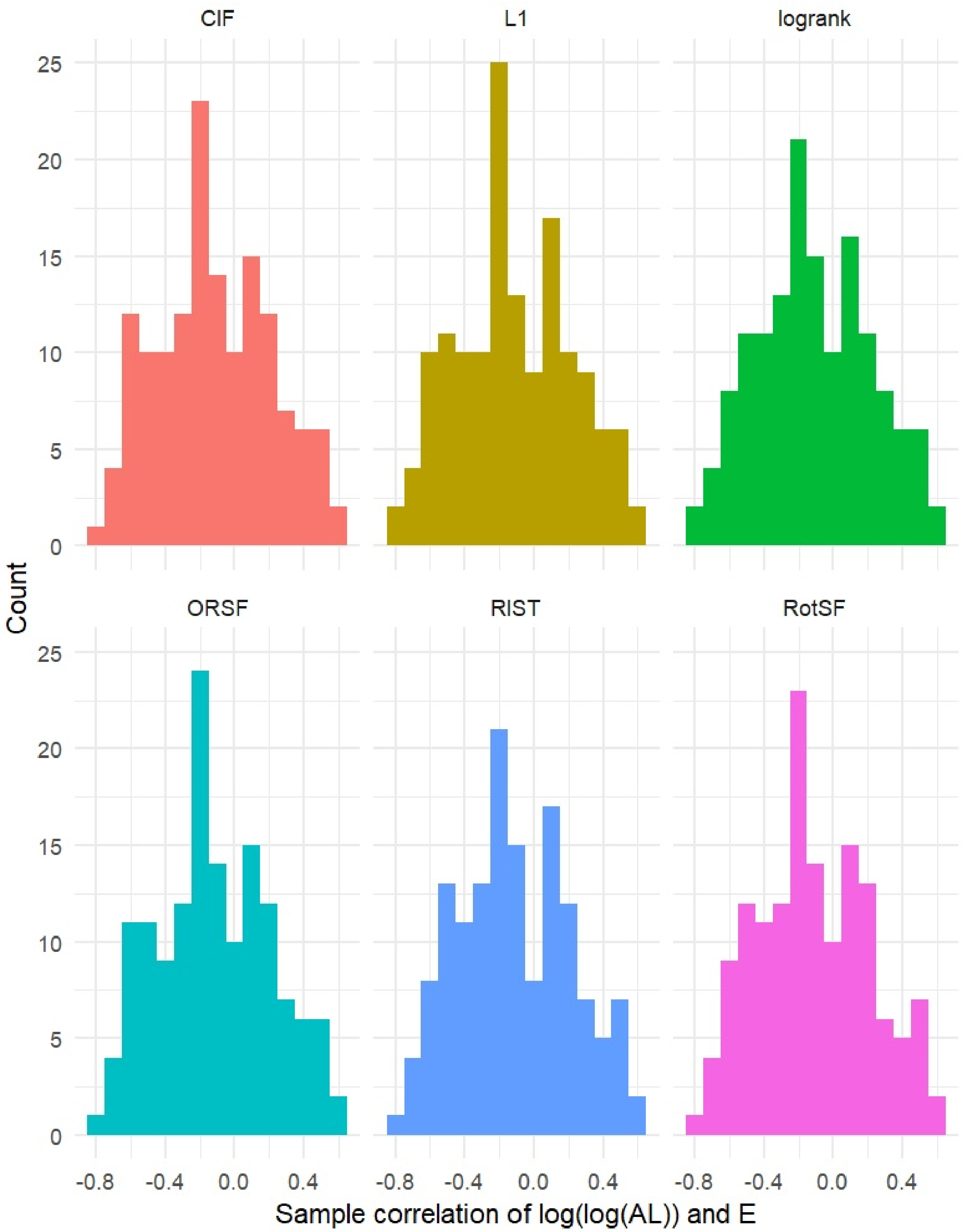

Figure 1: Histograms of the sample correlations of absolute loss and $E = 1 - C$ for each forest method within FLC

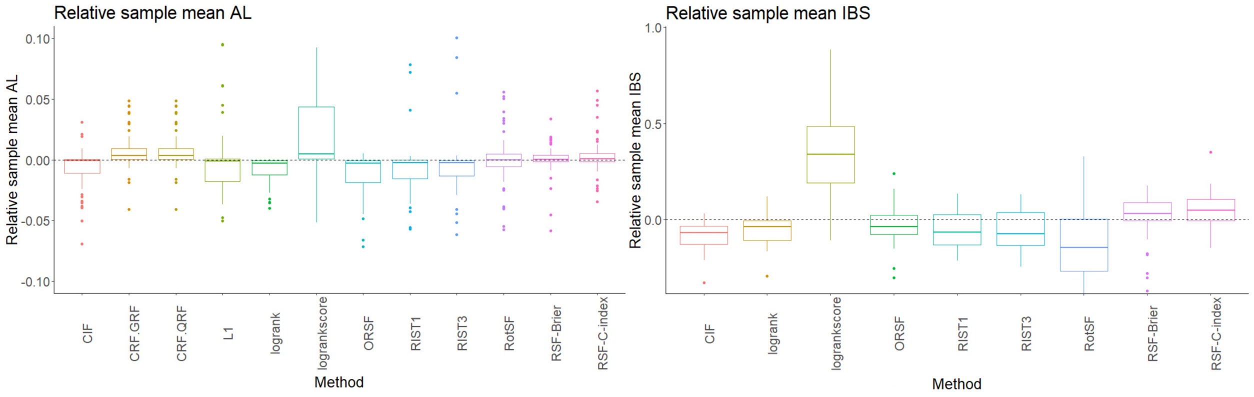

Figure 2: Relative sample mean absolute loss by method (left); Relative sample mean IBS by method

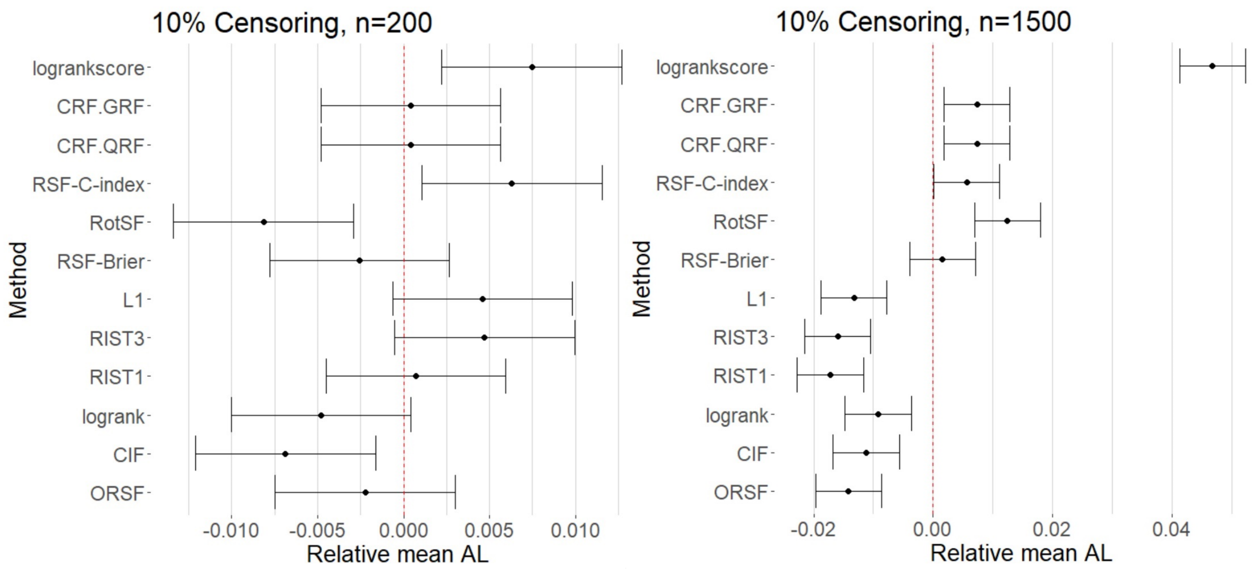

Figure 3: 95% simultaneous CIs of relative mean absolute loss (AL) for all methods at `SampleSize`=200 (left) and `SampleSize`=1500 (right) at `Censoring`=10%

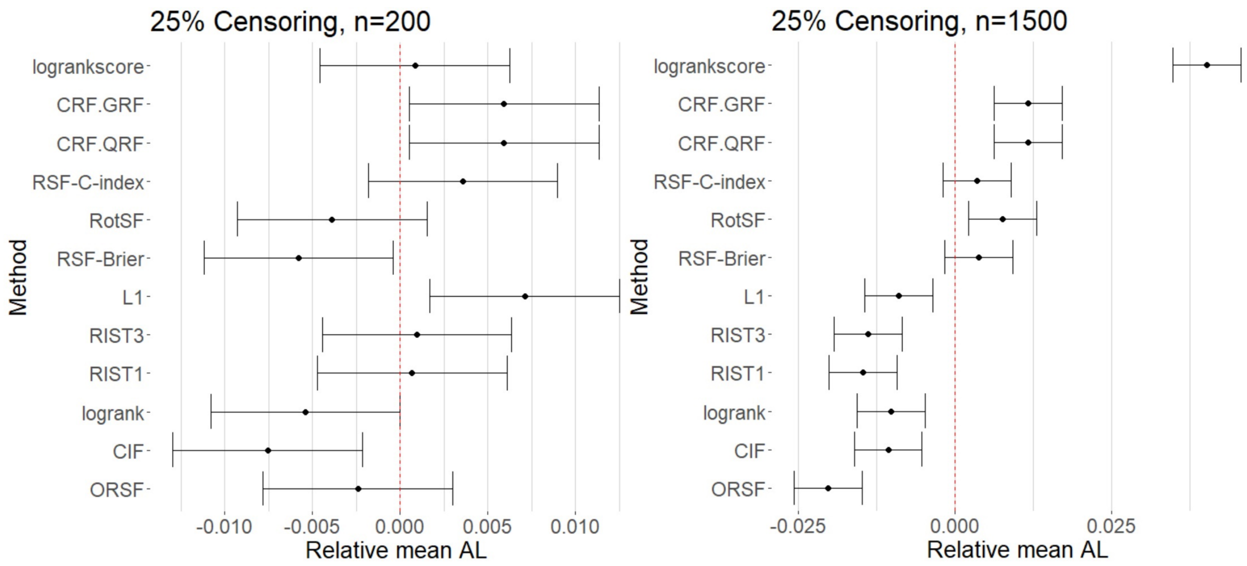

Figure 4: 95% simultaneous CIs of relative mean absolute loss (AL) for all methods at `SampleSize`=200 (left) and `SampleSize`=1500 (right) at `Censoring`=25%

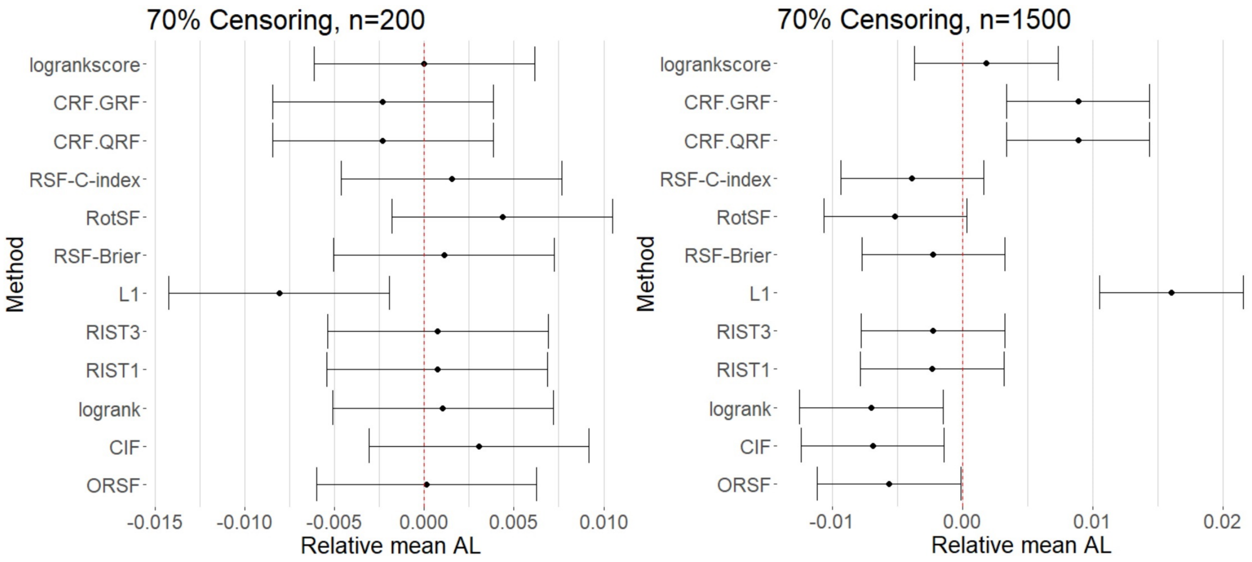

Figure 5: 95% simultaneous CIs of relative mean absolute loss (AL) for all methods at `SampleSize`=200 (left) and `SampleSize`=1500 (right) at `Censoring`=70%

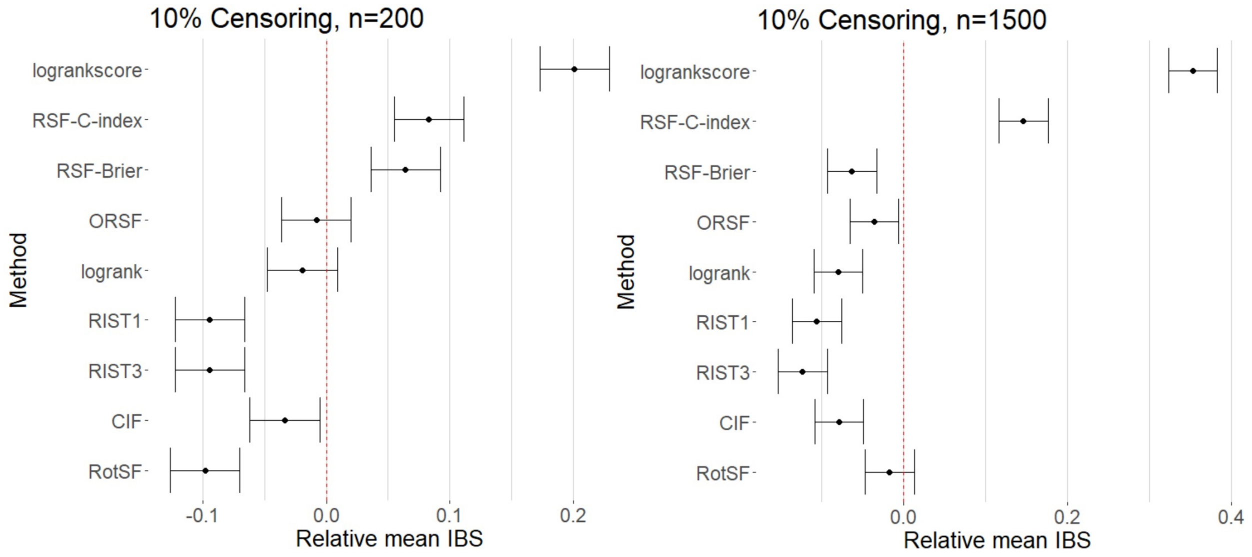

Figure 6: 95% simultaneous CIs of relative mean IBS for all methods at `SampleSize`=200 (left) and `SampleSize`=1500 (right) at `Censoring`=10%

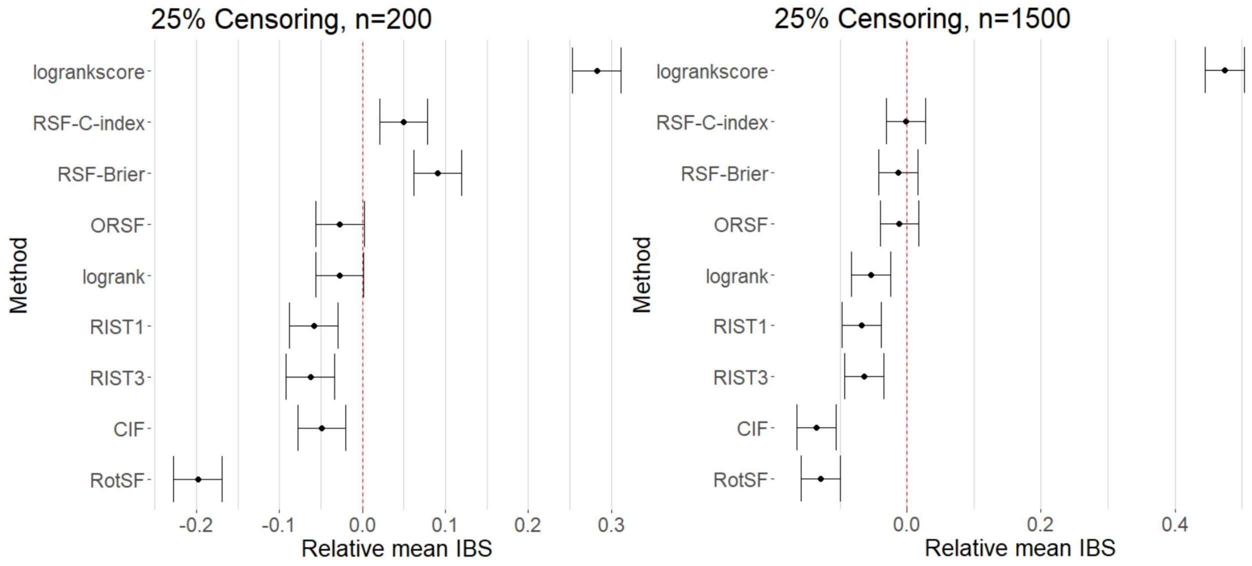

Figure 7: 95% simultaneous CIs of relative mean IBS for all methods at `SampleSize`=200 (left) and `SampleSize`=1500 (right) at `Censoring`=25%

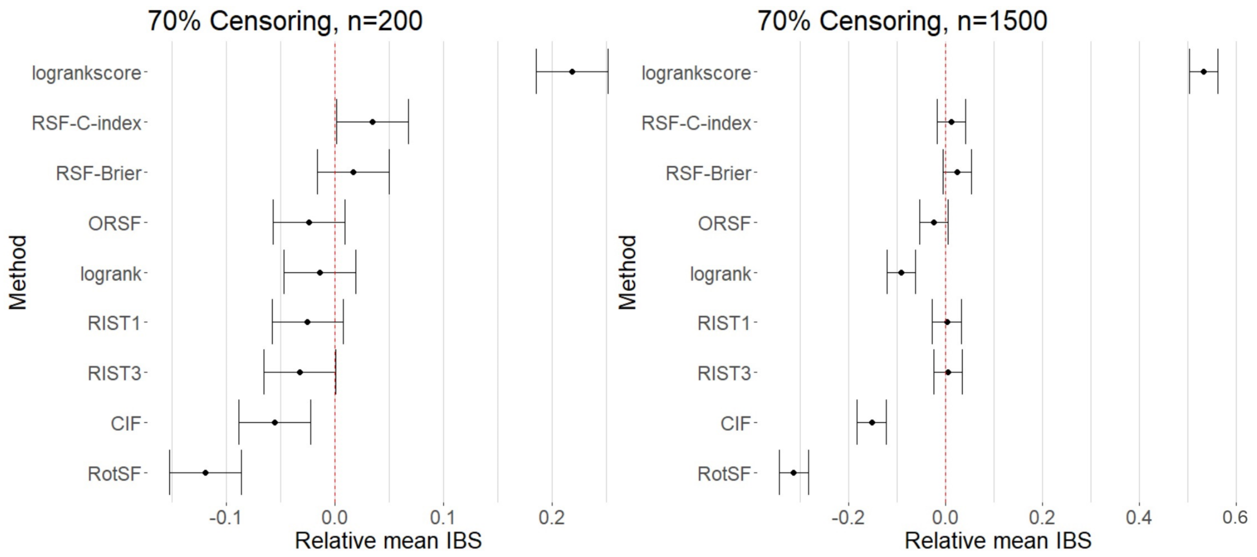

Figure 8: 95% simultaneous CIs of relative mean IBS for all methods at `SampleSize`=200 (left) and `SampleSize`=1500 (right) at `Censoring`=70%
